# *Aegilops sharonensis* genome-assisted identification of stem rust resistance gene *Sr62*

**DOI:** 10.1038/s41467-022-29132-8

**Published:** 2022-03-25

**Authors:** Guotai Yu, Oadi Matny, Nicolas Champouret, Burkhard Steuernagel, Matthew J. Moscou, Inmaculada Hernández-Pinzón, Phon Green, Sadiye Hayta, Mark Smedley, Wendy Harwood, Ngonidzashe Kangara, Yajuan Yue, Catherine Gardener, Mark J. Banfield, Pablo D. Olivera, Cole Welchin, Jamie Simmons, Eitan Millet, Anna Minz-Dub, Moshe Ronen, Raz Avni, Amir Sharon, Mehran Patpour, Annemarie F. Justesen, Murukarthick Jayakodi, Axel Himmelbach, Nils Stein, Shuangye Wu, Jesse Poland, Jennifer Ens, Curtis Pozniak, Miroslava Karafiátová, István Molnár, Jaroslav Doležel, Eric R. Ward, T. Lynne Reuber, Jonathan D. G. Jones, Martin Mascher, Brian J. Steffenson, Brande B. H. Wulff

**Affiliations:** 1 John Innes Centre, Norwich Research Park, Norwich, NR4 7UH UK; 2 Plant Science Program, Biological and Environmental Science and Engineering Division, King Abdullah University of Science and Technology (KAUST), Thuwal, 23955-6900 Saudi Arabia; 3 KAUST Center for Desert Agriculture, King Abdullah University of Science and Technology, Thuwal, 23955-6900 Saudi Arabia; 4 Department of Plant Pathology, University of Minnesota, St. Paul, MN 55108 USA; 5 The Sainsbury Laboratory, University of East Anglia, Norwich, NR4 7UK UK; 6 Institute for Cereal Crops Improvement, Tel Aviv University, Tel Aviv, Israel; 7 School of Plant Sciences and Food Security, Tel Aviv University, Tel Aviv, Israel; 8 Department of Agroecology, Aarhus University, Forsøgsvej 1, DK-4200 Slagelse, Denmark; 9 Leibniz Institute of Plant Genetics and Crop Plant Research (IPK), Seeland, Germany; 10 Center of integrated Breeding Research (CiBreed), Department of Crop Sciences, Georg-August-University, Von Siebold Str. 8, 37075 Göttingen, Germany; 11 Department of Agronomy, Kansas State University, Manhattan, KS 66506 USA; 12 Crop Development Centre, Department of Plant Sciences, University of Saskatchewan, Saskatoon, SK Canada; 13 Institute of Experimental Botany of the Czech Academy of Sciences, Centre of the Region Haná for Biotechnological and Agricultural Research, 779 00 Olomouc, Czech Republic; 14 2Blades Foundation, Evanston, IL USA; 15 German Centre for Integrative Biodiversity Research (iDiv) Halle-Jena-Leipzig, Deutscher Platz 5e, 04103 Leipzig, Germany; 16 Present Address: Syngenta Flowers, Downers Grove, IL 60515 USA; 17 Present Address: Leibniz Institute of Plant Genetics and Crop Plant Research (IPK)., Seeland, Germany; 18 Present Address: Agricultural Institute, Centre for Agricultural Research, ELKH, Martonvásár, 2462 Hungary; 19 Present Address: AgBiome, Inc., Research Triangle Park, NC 27709 USA; 20 Present Address: Alliance Management at Enko Chem, 62 Maritime Dr, Mystic, CT 06355 USA

**Keywords:** Plant evolution, Molecular engineering in plants, Agricultural genetics, Biotic

## Abstract

The wild relatives and progenitors of wheat have been widely used as sources of disease resistance (*R*) genes. Molecular identification and characterization of these *R* genes facilitates their manipulation and tracking in breeding programmes. Here, we develop a reference-quality genome assembly of the wild diploid wheat relative *Aegilops sharonensis* and use positional mapping, mutagenesis, RNA-Seq and transgenesis to identify the stem rust resistance gene *Sr62*, which has also been transferred to common wheat. This gene encodes a tandem kinase, homologues of which exist across multiple taxa in the plant kingdom. Stable *Sr62* transgenic wheat lines show high levels of resistance against diverse isolates of the stem rust pathogen, highlighting the utility of *Sr62* for deployment as part of a polygenic stack to maximize the durability of stem rust resistance.

## Abstract (web summary)

*Aegilops sharonensis* is a wild diploid relative of wheat. Here, the authors assemble the genome of *Ae. sharonensis* and use the assembly as an aid to clone the *Ae. sharonensis*-derived stem rust resistance gene *Sr62* in the allohexaploid genome of wheat.

## Introduction

Stem rust, caused by the fungal pathogen *Puccinia graminis* f. sp. *tritici* (*Pgt*), is one of the most important diseases of wheat worldwide. Stem rust outbreaks were once common, but programs to eradicate the alternate host of this heteroecious fungus, the common barberry (*Berberis vulgaris*), and breeding for disease-resistant wheat cultivars brought the disease under control across most of Europe and North America by the 1950s^1^. Epidemics continued to occur in Australia in the 1970s^2^ and South Africa in the 1980s^3^. Yet, it was the discovery of stem rust in Uganda in the 1998–1999 growing season on wheat lines carrying *Sr31*, a widely deployed and, until then, fully effective stem rust resistance gene, which marked a new era in stem rust epidemics^4^. The causal strain popularly known as Ug99 (race TTKSK on the basis of a widely accepted North American differential set of genotypes) and its derivatives subsequently spread throughout most of East and South Africa and into Yemen and Iran and evolved to overcome additional stem rust resistance genes, including *Sr24* and *Sr36*^5,6^. In 2012, a severe outbreak of stem rust not related to the Ug99 lineage occurred in Ethiopia in the widely cultivated wheat cultivar Digalu. The “Digalu” *Pgt* strain was subsequently detected across the Middle East^7^ and in some European countries, including Sweden, Denmark, Germany and the UK^8,9^. In 2016, a stem rust outbreak in southern Italy affected thousands of hectares of both durum and bread wheat^10^, while a separate outbreak in Western Siberia caused 30–40% yield losses across 1–2 million hectares^11^. Stem rust epiphytotics are predicted to become more common as climate change continues, favouring the northward spread of the fungus^12^. This outlook highlights the importance of developing new wheat varieties with broad-spectrum, durable resistance to stem rust.

So far, 58 distinct stem rust resistance genes have been designated in wheat^13^. Just over half are from bread wheat (*Triticum aestivum*), while the remainder were introgressed into wheat from wild and domesticated *Triticum* spp. (eight *Sr* genes), *Aegilops* spp. (10 *Sr* genes), rye (*Secale cereale*; four *Sr* genes), wheatgrass (*Thinopyrum* spp.; four *Sr* genes) and the grass *Dasypyrum villosum* (one *Sr* gene)^13^. Most *Sr* genes are pathogen- and race-specific, but *Sr55/Lr67*, *Sr5*7/*Lr34* confer slow-rusting multi-pathogen resistance^14,15^. Thirteen of the 58 designated *Sr* genes have been cloned, including *Sr13*^16^, *Sr21*^17^, *Sr22*^18^, *Sr26*^19^, *Sr33*^20^, *Sr35*^21^, *Sr45*^18^, *Sr46*^22^, *Sr50*^23^, *Sr55*/*Lr67*^14^, *Sr57*/*Lr34*^15^, *Sr60*^24^ and *Sr61*^19^. In addition, a race-specific stem rust resistance gene with the temporary designation *SrTA1662* was cloned from *Aegilops tauschii*^25^. Most race-specific *Sr* genes encode nucleotide-binding and leucine-rich repeat (NLR) proteins, except for *Sr60*, which encodes a tandem kinase^24^. The non-pathogen-specific resistance genes *Sr57*/*Lr34* and *Sr55*/*Lr67* encode a putative ATP-binding cassette (ABC) transporter and a hexose transporter, respectively^14,15^.

Five *R* genes encoding tandem kinases were cloned from various Poaceae species^26^. The first, *Rpg1* on chromosome arm 1HS in barley, confers stem rust resistance^27^. *Yr15* on chromosome arm 1BS, originally from emmer wheat (*Triticum turgidum* ssp. *dicoccoides*) and subsequently introgressed into bread wheat, confers broad-spectrum stripe rust resistance^28^. *Sr60* on chromosome arm 5AS in diploid wheat *Triticum monococcum* confers race-specific stem rust resistance^24^. *Pm24* on chromosome arm 1DS is a powdery mildew resistance gene from the Chinese wheat landrace Hulutou^29^, and the most recent addition, *WTK4* on chromosome arm 7DS, confers powdery mildew resistance in *Ae. tauschii* and synthetic hexaploid wheat derivatives^25^.

*Aegilops sharonensis* (genome constitution S^sh^S^sh^) is a wild diploid relative of wheat in the Sitopsis section found in present day Israel and southern Labanon^30^. The species possesses many traits of agricultural importance, including resistance to major diseases of wheat such as the rusts^31,32^. However, the presence of gametocidal genes in the genome of *Ae. sharonensis* that restrict interspecies hybridisation have hampered the introgression of its chromatin into wheat^33–37^. Indeed, of all the 264 designated resistance genes that have been introgressed into wheat, only three are from *Ae. sharonensis*, namely *Lr56*, *Yr38* and *Sr62*^13,34^.Therefore, the genetic potential of *Ae. sharonensis* remains largely untapped.

Here, we generate a reference-quality genome assembly of *Aegilops sharonensis* and clone the stem rust resistance gene *Sr62*, earlier designated as *Sr1644-1Sh*^38^. *Sr62* encodes a tandem protein kinase whose individual kinase domain homologues appear across the plant kingdom. We transform *Sr62* into the susceptible wheat cultivar Fielder and confirm the effectiveness of the gene against a range of geographically distinct *Pgt* isolates.

## Results

### Sequencing and assembly of the *Aegilops sharonensis* genome

*Ae. sharonensis* accession AS_1644 was previously used as the resistant parent in creating a recombinant inbred line population to map the stem rust resistance genes *Sr62* (*Sr1644-1Sh*) and *Sr1644-5Sh* and as a donor to introgress these genes into wheat cultivar Zahir^37,38^. To facilitate the cloning and characterisation of these genes, we generated a genome assembly of AS_1644. A line derived from this accession, which had been advanced through two generations of single-seed descent, contained residual heterogeneity of less than one single-nucleotide polymorphism (SNP) per 10 kb based on analysis of whole-genome shotgun (WGS) reads mapped to 1,440 conserved ‘benchmarking universal single-copy ortholog’ (BUSCO) genes. We utilized multiple technologies to sequence this inbred AS_1644 line (Supplementary Fig. 1) and assembled the genome using the TRITEX pipeline^39^. In brief, we performed Illumina sequencing-by-synthesis on WGS short-insert, long mate-pair (LMP), 10X linked read and Hi-C chromatin conformation capture libraries, as well as chromosome flow-sorted short-insert libraries (Supplementary Fig. 2; Supplementary Tables 1 and 2). We derived contig assemblies from the WGS short-insert libraries, scaffolded them sequentially with the LMP and 10X data, and connected them into chromosome pseudomolecules using the Hi-C data.

We obtained a scaffold assembly size of 6.7 Gb and a scaffold N50 and N90 of 12.3 and 1.1 Mb, respectively (Table 1). The assembly size of the chromosome pseudomolecules is 6.3 Gb, including 886 Mb of unfilled gaps (Table 1). The chromosome sizes range from 783 Mb (chromosome 1) to 1,022 Mb (chromosome 2). Unanchored scaffolds account for 420 Mb (Table 1). To assess the assembly quality, we performed BUSCO analysis. The assembly contains 96.5% complete BUSCOs and only 1.3% fragmented and 2.2% missing BUSCOs (Supplementary Fig. 3). The structural integrity of the pseudomolecules was supported by inter- and intrachromosomal Hi-C contact matrices (Supplementary Figs. 4, 5), its concordance with sequence data from flow-sorted individual chromosomes (Supplementary Fig. 6), and by collinearity with an *Ae. sharonensis* consensus genetic linkage map comprising 727 sequence markers and spanning 631 cM^38^ (Supplementary Fig. 7).

**Table 1:** *Aegilops sharonensis* AS_1644 v 1.0 genome assembly statistics.

Assembly characteristics	Values
Assembly size	6.7 Gb
^a^Scaffold N50	12.3 Mb
^a^Scaffold N90	1.1 Mb
Pseudomolecule size	6.3 Gb
Unfilled gaps	886 Mb
Chromosome 1S^sh^	783 Mb
Chromosome 2S^sh^	1022 Mb
Chromosome 3S^sh^	972 Mb
Chromosome 4S^sh^	827 Mb
Chromosome 5S^sh^	868 Mb
Chromosome 6S^sh^	807 Mb
Chromosome 7S^sh^	1016 Mb
Unassigned to a chromosome	420 Mb
Complete BUSCOs	0.965
Fragmented BUSCOs	0.013
Missed BUSCOs	0.022

^a^Scaffolds <1 kb were excluded.

Sequence comparison with high-confidence genes from the Chinese Spring A-subgenome^40^ identified 29,849 *Ae. sharonensis* candidate genes (84.5% of the total) at a cut-off of 90% sequence identity (Supplementary Table 3). This increased to 30,260 candidate genes (88.4%) after comparison to the D-subgenome and 30,626 (85.9%) after comparison to the B-subgenome. This analysis suggests that at least 30,626 high-confidence genes are present in our *Ae. sharonensis* genome assembly. We detected strong collinearity in the distal regions between the *Ae. sharonensis* and Chinese Spring subgenomes but disrupted collinearity in the centromeric regions (shown for the D-subgenome in Supplementary Fig. 8).

### Positional mapping of *Sr62*

**Fig. 1:**
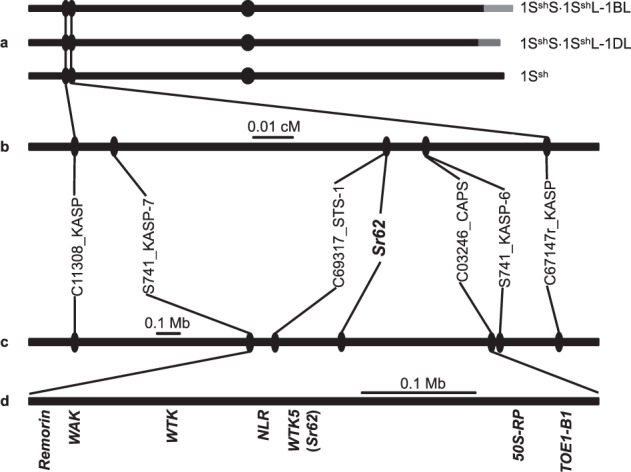
Positional mapping restricts *Sr62* to a 480 kb interval on chromosome 1S^sh^. **a** Wheat–*Ae. sharonensis* translocation chromosomes and *Ae. sharonensis* chromosome 1S^sh^. **b** Genetic map of the region harbouring *Sr62* on the short arm of *Ae. sharonensis* chromosome 1S^sh^. **c** Physical map of the region around *Sr62*. **d** Genes in the interval genetically delimiting the presence of *Sr62*. WTK is presumably an ortholog of *Pm24*^29^.

Resistance to stem rust was previously transferred into the wheat cultivar Zahir as a compensating Robertsonian translocation between *Ae. sharonensis* chromosome 1S^sh^ and the closely related wheat chromosome 1BL/1DL (1S^sh^S·1S^sh^L-1BL/1S^sh^S·1S^sh^L-1DL)^37^ (Fig. 1a). Independently of this, QTL mapping located *Sr62* on the short arm of chromosome 1S^sh^ in an *Ae. sharonensis* F_6_ recombinant inbred line population from a cross between accessions 2189 (susceptible) and 1644 (resistant)^38^. In the same population, a second stem rust resistance locus, *Sr1644-5Sh*, was localized to the long arm of chromosome 5S^sh^. To genetically separate these two *Sr* loci, we genotyped plants from F_2:3_ families and identified one plant, designated 803, that was heterozygous for markers diagnostic for *Sr62* on chromosome 1 and homozygous for a marker diagnostic of the susceptible haplotype at *Sr1644-5Sh* on chromosome 5 (Supplementary Fig. 9a; Supplementary Table 4). We phenotyped and genotyped 49 F_3:4_ plants derived from plant 803 and observed a 3:1 segregation ratio for resistance and susceptibility and congruency between resistance and PCR molecular markers diagnostic for accession 1644 on chromosome 1 (Supplementary Fig. 9b; Supplementary Table 4). These results indicated that we had genetically isolated *Sr62* and that the gene is dominant in *Ae. sharonensis*. Furthermore, the markers delimited the position of *Sr62* to a 12-cM interval between proximal marker C23635_CAPS and distal marker C25971_CAPS (Supplementary Fig. 9c; Supplementary Data 1 and Supplementary Tables 5–7).

To fine-map *Sr62*, we developed a large population segregating for *Sr62* by selfing F_4:5_ and F_5:6_ plants derived from plant 803 and heterozygous for the *Sr62* interval on chromosome 1. We genotyped 4,638 plants from this population with markers flanking *Sr62*, revealing 12 recombinants between C11308_KASP and C67147_KASP (Fig. 1b; Supplementary Fig. 9d; Supplementary Table 8). By generating more markers in this interval and phenotyping progeny of the 12 recombinants, we mapped *Sr62* to the region between markers S741_KASP-7 and C03246_CAPS (Supplementary Table 8), corresponding to a physical interval of 480 kb on the short arm of *Ae. sharonensis* chromosome 1 (Fig. 1c).

We performed RNA-Seq on AS_1644 and mapped the reads to the genome assembly. This identified seven transcribed genes in the interval with homology to the genes encoding a remorin family protein, a wall-associated kinase (WAK), two wheat tandem protein kinases (dubbed WTK5 and WTK), an NLR, a 50S  ribosomal protein (50S-RP) and a target of Eat1-B1 protein (TOE1-B1) (Fig. 1d).

### Identification of an *Sr62* candidate by EMS mutagenesis and RNA-Seq alignment

To identify *Sr62* among the candidate genes in the mapping interval, we performed EMS mutagenesis of Zahir-1644 wheat–*Ae. sharonensis* introgression lines, in which most of wheat chromosomes 1D or 1B were replaced by chromosome 1S^sh^ of *Ae. sharonensis* accession 1644^37^ (Fig. 1a). We mutagenized 3,025 Zahir-1644 seeds with 0.75% EMS. Eight or more seeds from the surviving 1649 M_2_ families were screened for susceptible mutants using the *Pgt* isolate Ug99 (race TTKSK). Thirty families segregating for resistance and susceptibility were identified and tested in the M_3_ generation, revealing 14 independent susceptible mutants. Genotyping-by-sequencing^41^ of Zahir, the two Zahir-1644 introgression lines and 10 EMS-derived mutants allowed us to rule out cross-contamination from other wheat cultivars for this mutant subset and to determine the translocation type, 1S^sh^S·1S^sh^L-1BL or 1S^sh^S·1S^sh^L-1DL (Supplementary Figs. 10–20).

**Fig. 2:**
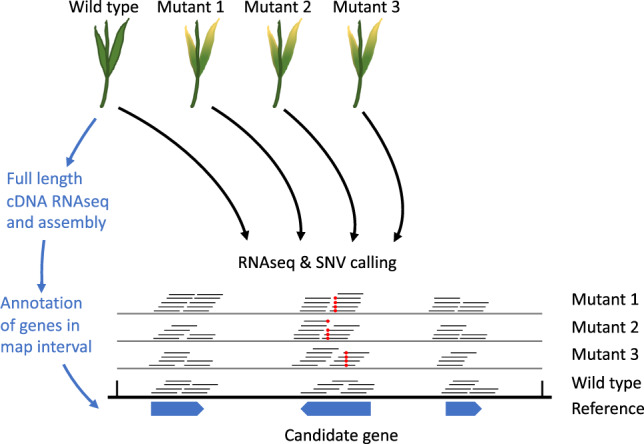
Candidate gene identification by mutagenesis and transcriptome sequencing (MutRNA-Seq). RNA-Seq reads from the wild-type parent and independently derived EMS mutants are mapped to a reference genome sequence. Annotated genes are inspected (within a mapping interval, if available) for a gene exhibiting a preponderance of single-nucleotide variants (SNVs, red dots) across the mutants.

**Fig. 3:**
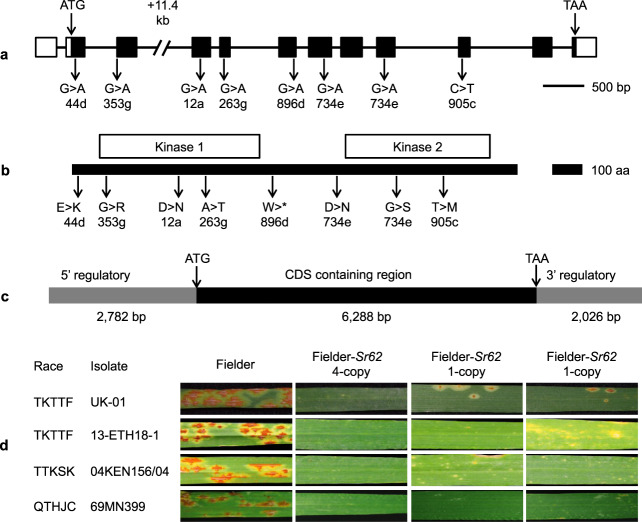
Functional validation of *Sr62* by EMS mutagenesis and transformation into wheat. **a** Structure of *Sr62*, with predicted nucleotide change caused by EMS-derived loss-of-function mutations. Boxes represent exons and lines represent introns with white boxes representing untranslated regions and black boxes representing the predicted open reading frame. The 11.4-kb portion of the third intron excluded from the binary construct is indicated. **b** Schematic representation of the *Sr62* protein, with the position of the two protein kinase domains and the predicted amino-acid changes caused by the EMS mutations indicated. **c** The *Sr62* sequence used for transformation of wheat cultivar Fielder. CDS, coding DNA sequence. **d** Reactions of three homozygous independent transgenic lines to four *Pgt* isolates. The copy number of the hygromycin selectable marker in T_0_ plants is indicated.

We constructed a full-length cDNA library for AS_1644, sequenced this library on the Illumina sequencing platform (generating 98 million 150-bp paired-end reads) and assembled these data to obtain transcripts of the seven genes in the mapping interval. In parallel, we sequenced the leaf transcriptomes of Zahir-1644 and the 14 mutants by generating ≥91 million 150-bp paired-end RNA-Seq reads per sample. We mapped the mutant RNA reads to the transcripts of the seven genes. This procedure, which we termed MutRNA-Seq (Fig. 2), identified eight point-mutations in WTK5 among seven of the 14 mutants. All of the mutations were G/C-to-A/T transition mutations, typical of EMS^42^. We predicted the open reading frame of WTK5 and found that seven of the mutations introduced non-synonymous changes, whereas one introduced an early stop codon (Fig. 3a, b). In contrast, WTK, the NLR, the 50 S ribosomal protein and TOE1-B1 had two or no mutations. For the WAK and remorin genes, the expression levels were too low to reliably call mutations (Supplementary Table 9 and Supplementary Data 2).

We developed a formula (function (4), see ‘Methods’ section) to test if WTK5 was the candidate among the seven genes. We graphically displayed the minimum number of mutants required to successfully identify a candidate gene by mutational genomics as a function of the number of genes investigated (Supplementary Fig. 21). We considered typical scenarios encountered in mutational genomics studies, such as when scrutinizing all genes in a discrete mapping interval (e.g., ten genes); a whole gene family (e.g., all 3,200 NLR loci in hexaploid wheat^43^); a whole chromosome, as obtained by chromosome flow sorting (i.e., ~5100 genes); or all ~107,000 genes in the hexaploid wheat genome^40^. This analysis indicated that the minimum number of independent mutants required to identify a candidate gene with a 2000-bp coding DNA sequence (CDS) at *p* = 0.01 is 3, 5, 5, or 6, respectively, for the abovementioned scenarios. This increases to 8, 16, 16, and 20 mutants, respectively, when dealing with two complementation groups. For our *Sr62* mapping interval, which contains seven genes, the probability of obtaining 7 out of 14 mutants with a mutation in the sequence of at least one of the seven genes being investigated based on the *WTK5* CDS (2,223 bp) would be 9.0 × 10^–5^. In other words, *WTK5* emerged as the best candidate among the seven genes, prompting further functional analysis.

### The *Sr62* candidate confers stem rust resistance in transgenic wheat

By mapping the leaf transcriptome RNA-Seq reads to the genome assembly of *Ae. sharonensis*, we predicted that the *WTK5* transcript spans 18,384 bp and contains 11 introns (Fig. 3a; Supplementary Fig. 22). To engineer a binary construct containing *WTK5*, primer pairs (Supplementary Table 10) were designed to amplify two parts of the genomic DNA sequence that cover most of the native gene, including 2.8 kb of putative promoter sequence 5′ of the predicted start codon and 2.0 kb of putative terminator region 3′ of the predicted stop codon but excluding 11.4 kb of the middle of the 12.4-kb intron (Fig. 3a). The two PCR products were separately cloned and combined into a binary vector via three-way ligation: the resulting recombined *WTK5* spans 11.9 kb (Fig. 3c). The construct was verified by Sanger sequencing and transformed into wheat cultivar Fielder. We obtained three independent primary transgenic lines (T_0_), which, based on qRT-PCR of the selectable marker, were predicted to contain one copy of the transgene (two lines) or four copies of the transgene (one line). We advanced these hemizygous lines to the next generation to obtain homozygous lines. All three lines conferred resistance to *Pgt* stem rust races TTKSK (isolate 04KEN156/04 from Kenya), TKTTF (isolate 13-ETH18-1 from Ethiopia), TKTTF (isolate UK-01 from the UK) and QTHJC (isolate 69MN399 from the US) (Fig. 3d), whereas the null plants were all as susceptible as the parent cv. Fielder (Supplementary Fig. 23). We also tested the line with four copies of the selectable marker against an additional eight *Pgt* isolates/races from Israel (three isolates), Italy (three isolates), Kenya (one isolate) and Ethiopia (one isolate) and found high levels of resistance (Supplementary Table 11; Supplementary Fig. 24).

### Both protein kinase domains are required for *Sr62* function

We mapped the mutations in *Sr62* relative to the two protein kinase domains. The seven amino acid substitutions are spread evenly throughout the predicted amino acid sequence, with three mutations in the Kinase 1 domain and two in Kinase 2 (Fig. 3b). Moreover, the premature stop codon mutant 896d leads to a predicted truncated protein lacking Kinase 2. Based on sequence alignment to previously characterized plant protein kinases, *Sr62* is predicted to encode a protein with two serine/threonine kinase domains (Supplementary Fig. 25). In mutant 353g, a conserved glycine residue at amino acid position 57 was substituted with an arginine proximal to the conserved ATP-binding site of Kinase 1, whereas the aspartic acid-to-asparagine substitution at amino acid position 177 (mutant 12a) is located in the catalytic site of Kinase 1 (Supplementary Data 3 and 4). Moreover, the substitutions at positions 57 and 177 are predicted to be intolerant (Supplementary Table 12). We developed a 3D model of the structure of *Sr62* using the Phyre2 web portal^44^ and CCP4MG^45^ and mapped these two amino acid substitutions onto the model. Interestingly, the Aspartate177Asparagine mutation is in a critical residue for kinase function (Supplementary Fig. 26). In active kinases, this Aspartate is involved in binding the phosphate of ATP, and acts as the catalytic residue^46^ functioning as a base acceptor for the proton transfer (Supplementary Fig. 26). The PDB coordinates of the comparative model can be found in the source data of Supplementary Fig. 26. We searched Sr62 for the presence of eight conserved amino acids that are diagnostic for protein kinases^47^. All eight of these amino acids are found in Kinase 1, whereas five out of the eight are present in Kinase 2. Based on this analysis, *Sr62* has a predicted kinase-pseudokinase structure, similar to *Pm24*. However, our mutant analysis suggests that both protein kinase domains are required for *Sr62* function. One of the amino acid substitutions (G539S) in Kinase 2 is predicted to be intolerant (Supplementary Table 12).

### Sequence relationship between *Sr62* and other tandem kinases in grasses

**Fig. 4:**
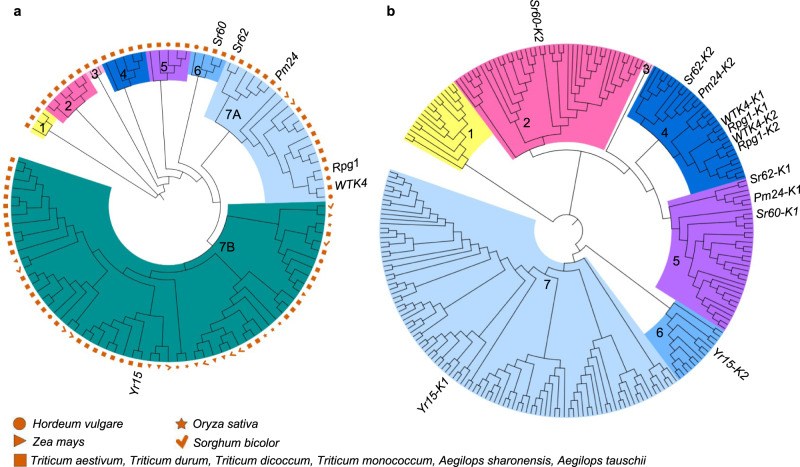
Phylogenetic relationship between tandem kinases from cereal crop and wild grasses. A total of 99 predicted tandem kinases were retrieved from the genomes of bread wheat, durum wheat, maize, barley, sorghum, rice, *Ae. tauschii* and *Ae. sharonensis*, along with the five cloned tandem kinase disease resistance genes. Phylogenetic clades and subclades are indicated by different colours and labelled with numbers. **a** Phylogeny based on the whole tandem kinase coding sequence. **b** Phylogeny based on the individual protein kinase domain coding sequences.

To explore the relationship between *Sr62* and other tandem kinases in major cereal crop species and wild grasses, we performed phylogenetic analysis of tandem kinases in hexaploid wheat (*Triticum aestivum*), durum wheat (*Triticum durum*), barley (*Hordeum vulgare*), rice (*Oryza sativa*), sorghum (*Sorghum bicolor*), maize (*Zea mays*) and the wild wheat relatives *Ae. tauschii* and *Ae. sharonensis* (Fig. 4a). The resistance genes *Sr62*, *Pm24*, *Rpg1*, *WTK4* and *Yr15* belong to the most populous clade, while *Sr60* sits in a small and separate, but closely related, clade (Fig. 4a). The closest neighbour of *Sr62* is *Pm24*, which appears to be a wheat chromosome 1D orthologue of *Ae. sharonensis WTK* situated 193 kb away from *Sr62* (Fig. 1d). However, the identity between *Sr62* and *Pm24* is low (<65%) at both the CDS and amino-acid sequence levels (Supplementary Table 13). We next generated a phylogenetic tree with the two protein kinase domains from each tandem kinase separated from each other. In this tree, the two domains of *Rpg1* and *WTK4* are located near each other in Clade 4 (Fig. 4b). By contrast, the two domains of *Sr62* and *Pm24* sit in different clades (Clades 4 and 5), but both *Sr62-K2* and *Pm24-K2* sit in Clade 4, and both *Sr62-K1* and *Pm24-K1* sit in Clade 5. The two domains of *Sr60* and *Yr15* are also in distinct clades (Clades 6 and 7 for *Yr15* and Clades 2 and 5 for *Sr60*). Based on pair-wise alignment using the Needleman–Wunsch method^48^, the two *Sr62* kinase domains share 51% identity. None of the five cloned tandem kinase genes contain kinase domains with more than 80% nucleotide identity.

Using a Hidden Markov Model-based classification approach for protein kinases developed by Lehti-Shiu & Shiu^49^, we investigated the individual protein kinase domains in Rpg1, Pm24, Yr15, Sr60, WTK4 and Sr62. Sr62 has a DLSV-DLSV configuration as do Rpg1 and Pm24, whereas the Sr60 protein kinase domains belong to the DLSV (domain 1) and CR4L (domain 2) subfamilies, and the Yr15 domains belong to the WAK (domain 1) and RLCK-VIII (domain 2) subfamilies.

### The *Sr62* kinase domains are of an ancient origin

We performed coding sequence homology analysis of *Sr62* using Gramene, a resource for comparative genomics across the plant kingdom^50,51^. Homologues were detected in five phyla: Tracheophyta, Bryophyta, Marchantiophyta, Chlorophyta and Rhodophyta (Supplementary Data 5). Thus, the homologues of the *Sr62* kinase domains are present in species ranging from unicellular green and red algae to mosses, liverworts and crop species including cereals (wheat, barley, rice, maize and sorghum), brassicas, potato, tobacco and coffee. The number of homologues varies widely, from three in the red alga *Chondrus crispus* to 65 in the wild tobacco *Nicotiana attenuata*. The *Triticum* species *T*. *aestivum* (hexaploid), *T*. *durum* (tetraploid), *Triticum dicoccum* (tetraploid) and *Triticum urartu* (diploid) have 26, 34, 19 and 21 homologues, respectively (Supplementary Data 5). These data suggest that the origin of the *Sr62* kinase domains predates the diversification of plants.

### The synteny around *Sr62* is specific to closely related grasses

**Fig. 5:**
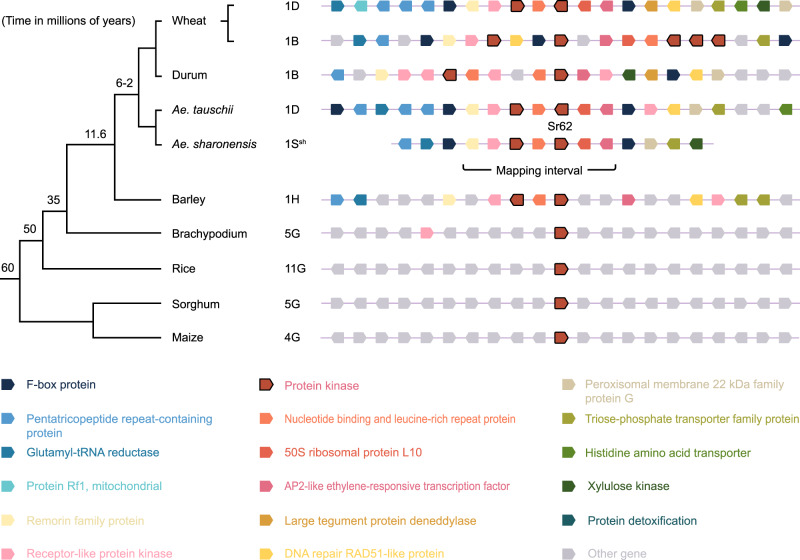
Synteny around *Sr62*. Genomic regions containing genes orthologous to *Sr62* along with surrounding genes reveal micro-synteny. The syntenic block is well conserved within the *Triticum* spp., *Aegilops* spp., and barley, but appears to be absent from *Brachypodium*, rice, sorghum and maize. The synteny alignment was generated through Gramene, except for *Ae. sharonensis*, which was added manually.

We conducted synteny analysis extending to ten genes on either side of *Sr62* in *Ae. sharonensis* and compared this region across nine Poaceae genomes spanning 60 million years of evolution^52^. The synteny block contains an F-box protein, a glutamyl-tRNA reductase, a pentatricopeptide repeat-containing protein, a remorin family protein, a protein kinase, an NLR and a TOE1-B1 (Fig. 5). The synteny is well conserved within the *Triticum* and *Aegilops* species, and to a lesser extent with barley, where it has undergone extensive rearrangements relative to *Triticum* and *Aegilops*. However, the block appears to be absent from *Brachypodium*, rice, sorghum and maize, suggesting that it arose between 11.6 and 35 million years ago^52^ (Fig. 5).

## Discussion

We cloned the *Ae. sharonensis* major-effect, dominant stem rust resistance gene *Sr62* and found that it encodes a tandem kinase. To date, more than 300 disease resistance genes have been cloned in plants, most of which (189 of 310) contain NLRs^53^. Only a few, from various Poaceae species, encode tandem kinases: *Rpg1*, *Yr15*, *Pm24*, *Sr60* and *WTK4*, all of which were identified in the Triticeae^24,25,27–29^. These five genes, as well as *Sr62*, contain two protein kinase domains. In *Rpg1* and *WTK4*, the two protein kinase domains are close to each other in the same phylogenetic clade (Fig. 4).

Based on sequence conservation of the key amino acid residues for protein kinase function in the two kinase domains, *Yr15* and *Pm24* have been classified as encoding tandem kinase-pseudokinases, *Sr60* as encoding a tandem kinase-kinase and *Rpg1* encoding a tandem pseudokinase-kinase gene^24^. Here, based on amino-acid alignment with plant kinases, we identified two absolutely conserved amino acids (residues 57 and 177) in the binding site and catalytic site of Kinase 1 of *Sr62* (Supplementary Fig. 26). Two EMS-induced susceptible mutants, 353g and 12a, have non-synonymous mutations that alter these two key residues (Supplementary Data 3 and 4), implying that the function of Kinase 1 of *Sr62* is critical for stem rust resistance. Another susceptible mutant (896d) carries an early stop codon resulting in a truncated protein predicted to only contain the Kinase 1 domain, suggesting that an intact Kinase 2 is also required for *Sr62* function.

For more than 20 years, the guard model has provided a useful framework for understanding the molecular mechanism and evolution of plant resistance genes^54,55^. According to this model, plant resistance proteins guard the pathogenicity targets (guardees) of pathogen effector molecules. The interaction of an effector with the pathogenicity target is detected by the guard, leading to a conformational change that triggers signalling, resulting in downstream defence responses. In NLR proteins, the C-terminal leucine-rich repeats provide the guarding function, while the N-terminal nucleotide-binding and coiled-coil or TIR domains confer the signalling capacity^56–58^. In the absence of a resistance gene, the interaction between guardee and effector protein promotes pathogen growth, resulting in a susceptible phenotype. All three interactors—guard, guardee and effector—are subject to diversifying selection, but for the guardee, this can be constrained by the requirement to maintain cellular function. Duplication of the guardee can release it from this constraint and provide a ‘decoy’ for the effector^59^. Early experimental support for the guard hypothesis came from the study of *Arabidopsis thaliana* RIN4, which is guarded by the NLRs RPM1 and RPS4 and targeted by the bacterial effectors AvrRpm1 and AvrRpt2^55,60–62^, and tomato Pto, a serine/threonine protein kinase that is guarded by the NLR protein Prf and targeted by the bacterial effector avrPto^55,63,64^. Somewhat unusually, the *Pto* gene acts genetically as the resistance gene and is part of a complex of six paralogues within a 60-kb region within which *Prf* is embedded^65^. In *A. thaliana*, the PBL2 (kinase)–RKS1 (pseudokinase)–ZAR1 (NLR) complex triggers immunity upon detection of the *Xanthomonas campestris* effector AvrAC^66^.

Perhaps like the Pto protein kinase or the PBL2/RKS1 kinase/pseudokinase, *Sr62* is also a pathogenicity target guarded by an NLR. In our EMS mutational genomics experiment targeting *Sr62*, only seven of 14 susceptible mutants carried non-synonymous or missense mutations in the tandem kinase (*Sr62*). This suggests that we obtained second-site mutations in one or multiple genes required for *Sr62* function. Likewise, EMS mutagenesis of *Pm24* (which is syntenic to *WTK* in the *Sr62* haplotype) resulted in 11 mutations in *Pm24* out of 26 susceptible mutants^29^. This propensity for second-site mutations is unusual; typically, mutagenesis screens targeting major dominant *R* genes yield <20% second-site suppressors^18^. Identification of the second-site suppressors of *Pm24* and *Sr62* could provide insight into the mechanism of wheat tandem kinase resistance genes. *Sr62* and *Pm24* both lie adjacent to an NLR gene (Fig. 1). The NLR adjacent to *Pm24* did not confer powdery mildew resistance when transformed into a susceptible wheat cultivar^29^; however, this does not exclude the possibility that this physically linked NLR is involved in *Pm24* function. We identified two EMS mutations in the NLR next to *Sr62* (out of the 14 susceptible mutants) of which one was non-synonymous. Further work is required to determine whether the linked NLR functions in concert with the *Sr62* and *Pm24* tandem kinases in a mechanism similar, for example, to the requirement of tomato *Prf* (NLR) for *Pto* function^63–65^. Alternatively, *Sr62* could directly perceive the presence of its corresponding effector and activate a downstream signal cascade to confer resistance independent of the involvement of any NLR, as has been proposed for other WTK proteins^26^. In this model, the pseudokinase acts as an effector decoy target that works in concert with the active kinase to initiate defense signalling^26^.

We found that the protein kinase domains in Sr62 belong to the Pelle/DLSV subfamily, which is present in Streptophytes, including angiosperms, gymniosperms, bryophytes, and Steptretophyte algae^67^. The DLSV is highly expanded in angiosperms and is the largest protein kinase family and induced in response to biotic stress in *Arabidopsis thaliana*^67^. Our working hypothesis is that diverse plant pathogens have evolved effectors that target the DLSV protein kinase family in order to impair immune signalling. A major question is whether the tandem kinases are simply decoys^59^ or have a specific function in immunity (or another process).

To facilitate the practical exploitation of *Ae. sharonensis*, we developed a high-quality reference genome based on WGS sequencing and chromosome flow sorting. The *Ae. sharonensis* N50 scaffold size of 12.3 Mb compares well with those of other assembled Triticeae genomes, including barley (N50, 1.4 Mb)^68^, *Ae. tauschii* (N50, 11.4 Mb)^69^, durum wheat (N50, 6.0 Mb)^70^ and bread wheat (N50, 22.8 Mb)^40^. The assembly contains 96.5% complete BUSCOs, which is similar to the 95.7% of rice (Osativa v7.0)^71^, but higher than the 86.4% of the first version of the barley reference genome (Hvulgare IBSC_PGSB r1)^72^.

Using flow cytometry, Eilam et al. determined the nuclear DNA content (1C value) of *Ae. sharonensis* to be 7.52 pg^73^, which is equivalent to a genome size of 7.35 Gb. We constructed chromosome pseudomolecules covering 6.3 Gb. Compared to the assembled genome sizes of barley (4.98 Gb)^74^, *Ae. tauschii* (4.0 Gb)^69^ and *T. urartu* (4.79 Gb)^75^, this is the largest diploid Triticeae genome assembled to date. The 2S^sh^ and 7S^sh^ chromosomes are each physically longer than 1 Gb (Supplementary Fig. 5). Interestingly, the short arm of 7S^sh^ (as defined by synteny to other Triticeae) appears to be physically longer than the long arm (Supplementary Fig. 5).

The D-subgenome of hexaploid wheat had a slightly higher percentage of high-confidence genes that aligned with *Ae. sharonensis* (88.4%) than the A- and B-subgenomes (84.5% and 85.9%, respectively; Supplementary Table 3). This supports a closer relationship between *Ae. sharonensis* and the D-subgenome, as previously reported based on gene tree topologies^76,77^. Further supporting this close evolutionary relationship, extensive haplotype collinearities were observed between *Ae. sharonensis* and wheat D-subgenome chromosomes^78^. Therefore, future *Ae. sharonensis* introgressions into wheat should be directed to the D-subgenome to reduce the likelihood of genetic imbalance. However, gametocidal genes in *Ae*. *sharonensis* make it difficult to develop introgression lines for every chromosome^37,79^. Our *Ae*. *sharonensis* reference genome will support ongoing efforts to clone these gametocidal genes^35,80,81^, perhaps leading to tools for accelerating introgression of *Ae. sharonensis* chromatin into wheat. However, because of the hybridization barrier imposed by the gametocidal genes themselves, *Ae. sharonensis* remains a largely unexploited source of resistance to major diseases of wheat^30^. The reference genome presented here will aid in the molecular cloning of such resistance genes, allowing their incorporation into genetically modified (GM) polygene stacks.

Several recently developed technologies facilitate the cloning of disease resistance genes in plants^13^. NLR or WGS sequencing combined with association mapping have been successfully applied for the rapid cloning of *Sr46*, *SrTA1662* and *WTK4* in *Ae. tauschii*^22,25^. Mutagenesis combined with NLR sequencing (MutRenSeq) or chromosome sequencing (MutChromSeq) allowed the rapid cloning of the wheat resistance genes *Sr22*, *Sr26*, *Sr45*, *Sr61*, *Yr5a*, *Yr5b*, *Yr7* and *Pm2* and the barley gene *Rph1*^18,19,82,83^. Mutagenesis combined with mapping, chromosome flow sorting and de novo generation of a cultivar-specific reference-quality single-chromosome assembly facilitated the cloning of *Lr22a* and *Pm21*^84,85^. Here we used a combination of whole-genome de novo assembly, positional mapping and RNA sequencing of multiple EMS-derived mutants to clone *Sr62*. The assembled reference genome of *Ae. sharonensis* facilitated fine mapping without BAC libraries to delimit *Sr62* to a 480-kb interval based on screening 9,276 products of meiosis. We identified seven genes in the interval with homology to a remorin, WAK, WTK, NLR, WTK5, 50 S and TOE1-B1 gene. By applying RNA-Seq to 14 EMS-derived mutants, we identified *WTK5* as the best candidate. The transformation of *Sr62* into cv. Fielder confirmed that *Sr62* is sufficient to confer resistance to stem rust, indicating that MutRNA-Seq is a powerful tool for gene identification.

The effectiveness of mutational genomics approaches, including MutRenSeq^18,19^ and the MutRNA-Seq method developed in this study, relies on multiple factors, including (i) the number of genes controlling a phenotype, (ii) the delimitation of the target gene to a physical map interval, chromosome or gene family and (iii) the number of mutants obtained. To aid in the future experimental design of mutational genomics studies in hexaploid wheat, we calculated the minimum number of mutants required to confidently (at *p* = 0.01) identify the correct gene. When considering a discrete map interval, a gene family, a whole chromosome or indeed all the genes in the wheat genome, this ranges from three to six mutants, but it increases to eight to 20 mutants if two complementation groups are revealed by mutagenesis (Supplementary Fig. 21). In practice, most gene cloning studies require a combination of genetic mapping with gene knockout and/or gain-of-function experiments. Extending MutRNA-Seq to other plant species requires sexual reproduction and the ability to obtain mutants. Obtaining mutants is favoured by an agronomy that promotes a large seed set and facile plant husbandry. Polyploidy can also be considered an advantage. This makes it easier to achieve an effective mutagen dose without killing the emerging seedlings or causing sterility. In addition, polyploid plants typically tolerate a 4-fold higher mutation density compared to diploids^42^, which reduces the size of the population that needs screening.

Advances in genomics and bioinformatics, such as those described here, are fuelling an exponential growth in the discovery and cloning of disease resistance genes in wheat and its wild relatives^13^. This is providing exciting opportunities for engineering broad-spectrum and durable disease resistance into wheat. The current major obstacle is no longer technical but imposed by the socio-political stalemate on the acceptance of GM wheat^86^. However, in May 2021 the Argentine biotechnology company Bioceres Crop Solutions and the Latin American food production and high street franchise Havanna announced the marketing of Alfajores biscuits made from a GM wheat containing the *hahb-4* gene from sunflower conferring drought tolerance^87^. In November of the same year, the Brazilian National Biosafety Commission announced the approval of flour made from *hahb-4* wheat^88^. Hopefully this and other efforts will lower the barrier for introducing other GM traits into wheat, such as for disease resistance.

## Methods

### Phenotyping

The stem rust tests with TTKSK were carried out in the BSL-3 containment facility at the University of Minnesota. The greenhouse was maintained at 19–22 °C with a 14-h photoperiod and approximately 40% relative humidity. Plants were inoculated with *P. graminis* f. sp. *tritici* when the second leaf was fully expanded, 10–12 days after planting, at a rate of ~0.12 mg of spores per plant. The inoculated plants were then placed in mist chambers in the dark overnight at near 100% relative humidity and 22 °C for 16 h. After the 16-h incubation period in the dark, fluorescent lamps were turned on with the misting continuing for an additional 2 h. After that, the misters were turned off and the plants allowed to slowly dry under the lights. Plants were moved back to the greenhouse and then scored for reaction to stem rust 12 days later. The infection types (IT) were recorded using the Stakman scale^89^.

### DNA extraction and sequencing

Leaf tissue from a single plant of *Ae. sharonensis* accession 1644 (line number BW_24933) was collected at the 7-leaf stage, and the DNA was extracted using the CTAB method for large quantities of DNA^90^. PCR-free short insert libraries (450 bp and 800 bp) and long mate pair (MP) (3 kb and 6 kb) libraries were generated and sequenced at Novogene, while the 9 kb long MP library was generated and sequenced at the Roy J. Carver Biotechnology Center, University of Illinois.

DNA for Hi-C and 10X was extracted as outlined in Jupe et al.^91^ Briefly, nuclei were extracted from up to 1 g of fresh leaf tissue by homogenization in 10 ml of nuclei isolation buffer, filtered through cell strainers and separated from debris using a Percoll layer. The extracted nuclei were embedded in low-melting agarose plugs and exposed to lysis buffer with proteinase K and RNase A. DNA was released by digesting the agarose with Agarase enzyme (New England Biolabs, Ipswich, MA, USA) and analysed by pulsed-field gel electrophoresis.

High-molecular-weight (HMW) genomic DNA (>40 kb) was isolated from the agarose plugs using pulsed-field electrophoresis on a Blue Pippin instrument (Sage Science) following the high-pass protocol with minor modifications. The size and integrity of the recovered HMW DNA was evaluated on a Tapestation 2200 (Agilent) and quantified by fluorometry (Qubit 2.0). One 10X sequencing library was prepared following the Chromium Genome library protocol v2 (10X Genomics) and sequenced across two lanes of HiSeqX with 150-bp paired-end (PE) reads (Illumina), which produced ~827 million reads (~33× coverage). Long Ranger (10X Genomics) was used to generate FASTQ files for analysis.

### Flow cytometric analysis and sorting

Suspensions of mitotic metaphase chromosomes were prepared from root tips of *Ae. sharonensis* accession 1644 as described by Vrána et al.^92^ and Kubaláková et al.^93^. Briefly, root tip meristem cells were synchronized using hydroxyurea, accumulated in metaphase using amiprohos-methyl and mildly fixed in formaldehyde. Intact chromosomes were released by mechanical homogenization of 100 root tips in 600 µl ice-cold LB01 buffer. Microsatellites GAA and ACG were labelled on isolated chromosomes by fluorescence in situ hybridization in suspension (FISHIS) using 5′-FITC-GAA7-FITC-3′ and 5′-FITC-ACG7-FITC-3′ oligonucleotides (Sigma, Saint Louis, USA) according to Giorgi et al.^94^, and chromosomal DNA was stained by DAPI (4′,6-diamidino 2-phenylindole) at 2 µg/ml.

Chromosome analysis and sorting were performed using a FACSAria II SORP flow cytometer and sorter (Becton Dickinson Immunocytometry Systems, San Jose, USA). Bivariate flow karyotypes FITC vs. DAPI fluorescence were acquired for each sample, and chromosomes were sorted at a rate of 1500–2000 particles per second. Two batches of 25,000–76,000 copies of each chromosome (chromosomes 1Sh and 6Sh were sorted together) were sorted into PCR tubes containing 40 μl sterile deionized water.

The chromosome contents of flow-sorted fractions were estimated by microscopic observation of 1500–2000 chromosomes sorted into a 10-μl drop of PRINS buffer containing 2.5% sucrose^95^ on a microscopic slide. Air-dried chromosomes were labelled by FISH with probes for pSc119.2 repeat, GAAn microsatellite and 45S rDNA according to Molnár et al.^96^. At least 100 chromosomes were classified following the karyotype described by Zhang et al.^97^ and Badaeva et al.^98^ to determine the chromosome content of flow-sorted samples and to assign the populations observed on bivariate flow karyotypes to particular chromosomes.

Flow-sorted chromosome samples were treated with proteinase K, after which their DNA was purified and amplified by multiple displacement amplification (MDA) using an Illustra GenomiPhi V2 DNA Amplification Kit (GE Healthcare, Chalfont St. Giles, United Kingdom) as described by Šimková et al.^99^.

### Genome assembly

Chromosome-scale sequence assembly was performed using the TRITEX assembly pipeline as described by Monat et al.^39^. Libraries containing a ~450-bp insert size and sequenced with 250-bp paired-end reads (PE450 libraries) were merged with BBMerge^100^ and error-corrected with BFC^101^. Corrected libraries were used for iterative assembly with Minia3 (*k*-mer sizes: 100, 200, 250, 300, 350, 400, 450)^102^. Assembled unitigs were scaffolded with PE800, MP3, MP6 and MP9 data using SOAPDenovo^103^. Internal gaps in scaffolds were closed with GapCloser^103^. Chromosome-scale sequence scaffolds (pseudomolecules) were constructed with R scripts of the TRITEX pipeline using linkage information afforded by 10X linked-reads, Hi-C data, flow-sorting data and a genetic map^38^. As the genetic map of *Ae. sharonensis* was less dense than the POPSEQ maps of bread wheat^104^ and barley^105^, we modified the TRITEX workflow by adopting an iterative approach for pseudomolecule construction. In the first iteration, scaffolds were assigned to chromosomes using the Hi-C map and the genetic map as a guide. Hi-C data were then used to order scaffolds within chromosomes so that approximate chromosomal locations for most scaffolds were known. In the second iteration, this ordering of scaffolds was used to guide the construction of super-scaffolds with 10X linked reads, accepting only scaffold joins supported by both 10X reads and proximity in the Hi-C map. The 10X super-scaffolds were ordered along the chromosomes with Hi-C data. Flow-sorting data were used to corroborate Hi-C-based chromosome assignments and to correct errors. Finally, manual correction of chimeric scaffolds and refinements to the order and orientation were performed by visual inspection of Hi-C contact matrices. The gene content quality control analysis was conducted with BUSCO (v4.06, viridiplantae orthodb10).

### Initial *Sr62* mapping with 803 family

We developed several F_3:4_ families from the cross between accession 2189 (susceptible) and accession 1644 (resistant)^38^ by single-seed descent. One of these families, 803, segregated 3 resistant to 1 susceptible plant. Using this family, we developed a linkage map for *Sr62* and using 192 plants, delimited *Sr62* to between CAPS markers C24499_CAPS and C23635_CAPS (Supplementary Fig. 9).

Preliminary mapping was performed using genetic markers developed from the *Ae. sharonensis* 1644 genome that was based on 1.15e9 Illumina 100 bp paired-end reads^76^. WGS of *Ae. sharonensis* 2189 (7.6e8 Illumina 100 bp paired-end reads) were aligned to the *Ae. sharonensis* 1644 genome using BWA (BWA version 0.7.17). Single nucleotide variations were identified using BCFtools (version 1.2) and filtered using vcfutils.pl. False positive SNPs were minimized based on variations identified with self-alignment of *Ae. sharonensis* 1644 reads. A total of 2,608,758 SNPs were identified based on filtering for positions with unambiguous read support (i.e. homozygous variations). Using the barley consensus genetic map^106^, putative orthologs were identified in the *Ae. sharonensis* 1644 genome and SNVs selected for the development of Sequenom assays. Putative single nucleotide polymorphisms between *Ae. sharonensis* accessions 1644 and 2189 were extracted with 80 bp flanking sequence.

These sequences were used as templates for primer design using MassARRAY software v3.1 for the multiplexing of two 28 SNP assays (a total of 56 SNP assays). Sequenom genotyping was carried out at the Iowa State University Genomic Technologies Facility (Ames, IA, USA). All SNPs and WGS contig source information for Sequenom markers are detailed in Supplementary Data 1.

### High-resolution mapping of *Sr62*

We first screened 1,304 plants (2608 gametes) from the 803 family and identified 47 recombinants between the two flanking markers C24499_SBE and C23635_CAPS. These were restricted to six key recombinants with the STS marker C11837_CAPS. When the whole-genome shotgun sequencing and assembly became available, KASP (https://www.biosearchtech.com/Supplementaryport/education/kasp-genotyping-reagents/how-does-kasp-work) markers were developed by identifying SNPs between the genomic scaffold sequences of 1644 and those of the susceptible parent 2189^38^. Two of these markers, C122784_KASP and C2468909_KASP, were used to screen the progeny of 3,342 plants (6684 gametes) derived from heterozygous 803 family individuals. All markers (Supplementary Tables 5, 6, 7 and 10) were designed using Primer3 Input (http://bioinfo.ut.ee/primer3-0.4.0/).

### RNA extraction and *Sr62* annotation

Total RNA was extracted from *Ae. sharonensis* accession 1644 with a RNeasy Plant Mini Kit (Cat No./ID: 74904, Qiagen, Valencia, CA, USA) following the manufacturer’s protocol and digested with DNase (Roche). RNA-Seq was performed by Novogene. The RNA-Seq reads were trimmed with Trimmomatic (version 0.32, http://www.usadellab.org/cms/?page=trimmomatic). Hisat2^107^ (version 2.1.0) was used to map the short reads onto the *Sr62* reference sequence. The SAM output file was converted into a BAM file using SAMtools^108^ (version 1.8) (http://www.htslib.org/) and sorted according to their position in the reference and indexed for visualization by IGV (version 2.8.13, https://software.broadinstitute.org/software/igv/). The full-length cDNA library was constructed using SMARTer® PCR cDNA Synthesis kit (Cat. # 634926, Clontech/TaKaRa) and sequenced on Illumina platform. The sequence reads were assembled using CLC Assembly Cell v 5.0.0 (https://digitalinsights.qiagen.com/products-overview/discovery-insights-portfolio/analysis-and-visualization/qiagen-clc-assembly-cell/).

### Mutant development

To further determine the candidate gene for *Sr62*, we mutagenized 3,025 seeds of an introgression line containing *Sr62* derived from *Ae. sharonensis* accession 1644 in the hexaploid bread wheat (*T. aestivum*) cultivar Zahir background (Zahir-1644)^37^. Dry seeds were treated for 16 h with 200 ml of 0.75% EMS solution while being rolled on a Roller Mixer (Model SRT1, Stuart Scientific) to ensure maximum homogenous exposure of the seeds to the EMS. The excess solution was then removed, and the seeds were washed three times with 400 ml tap water. The M_1_ seeds were grown in the greenhouse, and the seeds of M_2_ families (single heads) were collected. Eight seeds per family were phenotyped with *Pgt* isolate 04KEN156/04, race TTKSK. The M_3_ seeds derived from susceptible M_2_ plants were also tested to confirm that the M_2_ susceptible plants were true mutants. To rule out seed contamination a subset of the mutants was verified using genotyping-by-sequencing (GBS)^109^.

GBS data from the background (Zahir), donor (*Ae. sharonensis*, accession 1644) and introgression lines were mapped to the reference sequence of Chinese Spring^40^ using BWA mem (version 0.7.12) with standard parameters^110^. Mappings were sorted and converted to mpileup format using SAMtools^108^ (version 0.1.19). The mpileup files were examined with a custom script to calculate the percentage of SNPs from the donor that were shared with the introgression line per given interval. Several interval lengths were tested; a clear signal was observed for 10 Mb.

### Germplasm

Seeds of wheat cultivar Zahir (DPRM0080), the Zahir-1644 introgression lines 1S^sh^S·1S^sh^L-1BL (DPRM0081) and 1S^sh^S·1S^sh^L-1DL (DPRM0092), six of the EMS-induced mutants (DPRM0082, DPRM0083, DPRM0084, DPRM0085, DPRM0086, DPRM0087), *Ae. sharonensis* accession 1644 (DPRM0088) and the three transgenic lines (DPRM0089, DPRM0090, DPRM0091) are available from the Germplasm Resources Unit, John Innes Centre, Norwich, UK (https://www.jic.ac.uk/research-impact/germplasm-resource-unit/).

### RNA mapping

Fourteen susceptible mutants derived from independent M_2_ families were selected for RNA-seq. Total RNA was extracted from 14-day-old seedlings of the susceptible mutants and the wild-type Zahir-1644 parent line. The raw reads from the 14 mutants were mapped to the CDS of the seven genes from the *Sr62* map interval using BWA^110^ (version 0.7.12) and SAMtools^108^ (version 1.8). One CDS in the interval was identified as having a single nucleotide mutation in seven of the 14 mutants (Supplementary Table 9). All identified mutations were G-to-A or C-to-T transition mutations, which are typical of EMS mutagenesis. The coverage of the remaining two genes (remorin and WAK) was too low to call mutations (Supplementary Table 9 and Supplementary Data 2). Reads per kilobases (RPK) and transcript per million (TPM) were calculated by BLASTing FASTA files converted from the assorted BAM files^108^ with the CDSs. The amino acid substitution tolerance/intolerance was analysed with THE web-based program SIFT^111^ (version 6.2.1) https://sift.bii.a-star.edu.sg/www/SIFT_seq_submit2.html).

### Formula development

We assume that there are *n* mutants in total and that *k* mutants have a mutation in the candidate gene CDS. The CDS length (in bp) is *L*_c_ and the mutation rate is *R*, and therefore the probability that the candidate CDS is present in a single mutant is *RL*_c_. Thus, the probability (*p*_c_) for the event, where *k* out of *n* mutants have a mutation in the candidate CDS, is a binomial distribution^112^, expressed as:

1 $${p_{c}}=\left({n}\atop{k}\right){\left({RL}_{c}\right)}^{k}{\left(1-\left({RL}_{c}\right)\right)^{n-k}}$$

If there were *m* genes being investigated (such as those in the genetic mapping interval of positional cloning) and their CDS were the same length as the CDS of the candidate gene, the probability (*p*) that the event occurs at one or more of the genes being investigated would be:

2 $$p=1-{\left(1-{pc}\right)}^{m}$$

Because CDS lengths vary among genes, it is necessary to introduce the concept of the effective number (*m*_*e*_). The effective number of the gene’s CDS relative to the candidate gene’s CDS is the total CDS length (*L*_i_) of the genes divided by the length (*L*_c_) of the candidate gene CDS.

3 $$m_{e}=\frac{{L}_{i}}{L_{c}}$$

Therefore, the probability of obtaining *k* out of *n* mutants with a mutation in the sequence of at least one of the genes being investigated is:

4 $$p=1-\left(1-\left(\frac{n}{k}\right)(RL_{c})^{k}(1-(RL_{c}))^{n-k}\right)^{\frac{L_{i}}{L_{c}}}$$

The CDS of most cloned plant disease resistance genes are ~2000 bp long^24,25,27–29^, while the average CDS length in wheat is 1,000 bp^75^. The probabilities displayed in Supplementary Fig. 21 were calculated based on the following assumptions: (i) the candidate gene has a 2000-bp CDS, (ii) every NLR has a 2000-bp CDS, (iii) the average CDS length of a gene on one chromosome or throughout the genome is 1000 bp, (iv) all mutants are assumed to have a mutation where the trait is controlled by one gene, and half of the mutants have a mutation where the trait is controlled by two genes. The probability for any candidate gene can be calculated given the specific genome mutation rate, candidate gene CDS length, total number of mutants, number of mutants with mutation in the CDS, and effective number of genes being investigated. In practice, the effective number can be approximated based on total number of genes being investigated by multiplying the average length (*L*_a_) of the gene CDS and then dividing by the candidate-gene CDS length.

5 $$m_{e}=\frac{{mL}_{a}}{L_{c}}$$

### Engineering *Sr62* binary construct

The 5′ and 3′ halves of *Sr62* containing 2782 bp of the putative promoter and 2026 bp of the putative terminator sequence, respectively, were PCR-amplified so as to leave out 11.4 kb of the third intron (Fig. 3a). The amplification was made with the high fidelity Q5 DNA polymerase (NEB, Ipswich, MA, USA) following the manufacturer’s instructions. The PCR products were purified with QIAquick PCR Purification kit (QIAGEN, LLC, Germantown, MD 20874, USA). The purified fragments were tailed with nucleotide A using *Taq* DNA polymerase and cloned into the pCR2.1 vector (TOPO PCR Cloning Kits-K202020, Thermo Fisher Scientific). The positive clones were multiplied and the plasmid DNAs were digested with two pairs of restriction enzymes, *Not*I + *Eco*RI (NEB, Ipswich, MA, USA) for the 5′ fragment and *Eco*RI + *Pme*I (NEB, Ipswich, MA, USA) for the 3′ fragment. The two digested fragments were gel purified and then ligated into the binary vector pGGG-AH-NotI/PmeI^113^ linearized with *Not*I and *Pme*I in a three-way ligation using T4 ligase (M0202S, NEB, Ipswich, MA, USA). A positive clone, pGGG-*Sr62*, was bulked and verified by Sanger sequencing. pGGG-*Sr62* is available from Addgene under the name pGGG-*Sr1644-1Sh*, accession number 164087.

### Wheat transformation

The binary construct pGGG-*Sr62* was used in Agrobacterium-mediated transformation of wheat cv. Fielder as described by Hayta et al.^113^. The copy number of the hygromycin selectable marker (as a proxy for the *Sr62* copy number) in T_0_ and T_1_ plants was determined by iDNA Genetics (Norwich, UK) using qPCR, as described in Bartlett et al.^114^.

### Evaluation of resistance to stem rust in transgenic wheat seedlings

The primary transgenic plants (T_0_) were tested for stem rust response with the UK-01 isolate^9^ and T_1_ plants were further tested with multiples isolates/races including TTKSK (isolate Ug99, 04KEN156/04 from Kenya), TKTTF (isolate 13-ETH18-1 from Ethiopia), TKTTF (isolate UK-01 from the United Kingdom), QTHJC (isolate 69MN399 from the USA), TKTSC (isolate #2079 from Israel), TTTTF (isolate #2127 from Israel), TTTTC (isolate #2135 from Israel), TTKTT (isolate KE184a/18, from Kenya), TKTTF (isolate ET11a/18, from Ethiopia), TKKTF (isolate, IT200a/18, from Italy), and TTRTF (isolate IT16a/18, from Italy) (Supplementary Table 11).

### Homology searching

BLAST analysis against TGAC CS42 v1 gene models was performed using the *Sr62* CDS as a query (http://eg37-plants.ensembl.org). The best hit (TRIAE_CS42_1BS_TGACv1_050726_AA0174710.1) was used for homology searching using Gramene, a comparative resource for plants^50,51^ (https://www.gramene.org/). The taxonomy for selected species/subspecies was retrieved from Taxonomy Browser (https://www.ncbi.nlm.nih.gov/Taxonomy/Browser/wwwtax.cgi?id=1437183). The taxonomy was organized by Kingdom, Phylum, class, order and species. Each selected species was blasted with the *Sr62* protein sequence or CDS at the NCBI webpage (https://blast.ncbi.nlm.nih.gov/Blast.cgi).

### Phylogenetic analysis of plant protein kinase domains

The CDS of the putative protein kinase genes previously extracted from the wheat cv. Chinese Spring reference genome (IWGSC, 2018) for phylogenetic analysis of *Yr15*^28^ were used as queries for BLAST analysis against the Chinese Spring, durum, barley, rice, sorghum and maize genomes (http://plants.ensembl.org/index.html). For *T. monococcum*, *T. dicoccum*, *Ae. tauschii* and *Ae. sharonensis*, only the cloned gene sequences were added to the phylogenetic trees. The sequence of the *Sr62* tandem kinase was determined based on the BLAST results with the CDS (https://blast.ncbi.nlm.nih.gov/Blast.cgi?PROGRAM=blastx&PAGE_TYPE=BlastSearch&LINK_LOC=blasthome, non-redundant protein sequence-nr). The retrieved CDS were used to perform BLAST analysis against the NCBI protein database (https://blast.ncbi.nlm.nih.gov/Blast.cgi?PROGRAM=blastx&PAGE_TYPE=BlastSearch&LINK_LOC=blasthome, non-redundant protein sequence-nr) and manually checked for the kinase domain. Genes with two or three complete protein kinase domains were selected for phylogenetic analysis. A total of 105 genes were used for analysis: 51 from Chinese Spring, 15 from durum, seven from barley, six from rice, 11 from sorghum, nine from maize and the six cloned Poaceae genes previously discussed (including *Sr62*). For kinase domain analysis, all 105 tandem kinase sequences were split into two or three kinase domain sequences and were used for phylogenetic analysis based on domain sequences. A phylogenetic tree (neighbour-joining tree) for whole genes and domains was computed with Clustal Omega (https://www.ebi.ac.uk/Tools/msa/clustalo/) and drawn with iTOL (https://itol.embl.de/). Hidden Markov Model classification was performed using hmmscan (v3.1b2). HMMs were obtained from the Supplementary Data set of Lehti-Shiu and Shiu^49^.

### 3D modelling

The 3D protein structure model was constructed with the program Phyre2^44^ (http://www.sbg.bio.ic.ac.uk/~phyre2/html/page.cgi?id=index) and visualized with CCP4MG^45^ (version 2.10.11).

### Micro-synteny analysis of *Sr62*

The *Sr62* CDS was used in a BLAST analysis to identify the putative orthologs in *T. aestivum*, *T*. *durum* (TRITD1Bv1G020740.1), *Ae. tauschii* (AET1Gv20143300, and *H. vulgare* (HORVU1Hr1G011730). No clear *Sr62* ortholog was detected in *T. aestivum* but a clear ortholog for *Pm24* could be identified. The positions of these best hits were used to extract the proximal and distal genes with Gramene^50,51^ (https://www.gramene.org/). For rice, *B. distachyon*, maize, and sorghum, the best hit with the putative barley *Sr62* ortholog, HORVU1Hr1G011730, was used for the synteny analysis. For *Ae. sharonensis*, the genes around *Sr62* were manually annotated based on the RNA-Seq and full-length cDNA data for accession 1644.

### Reporting summary

Further information on research design is available in the Nature Research Reporting Summary linked to this article.

## Supplementary information

Supplementary Information

Description of Additional Supplementary Files

Supplementary Data 1

Supplementary Data 2

Supplementary Data 3

Supplementary Data 4

Supplementary Data 5

Reporting Summary

## Data Availability

The datasets generated during and/or analysed during the current study are publicly available as follows. The sequence reads and the genome assembly were deposited in the European Nucleotide Archive (ENA) under project number PRJEB40322 and PRJEB40049, respectively. The RNA-Seq data for AS_1644, the full-length AS_1644 cDNA library, and Zahir-1644 introgression line wild type and its 14 mutants was deposited at NCBI under project number PRJEB47173 and the transcriptome assembly is available from e!DAL (10.5447/ipk/2021/21). The Zahir-1644 and mutant GBS data have been deposited in ENA under project number PRJEB46949. The *Sr62* gene and transcript sequence were deposited in NCBI Genbank under accession number MZ826707. The following public databases/datasets were used in the study: Chinese Spring reference genome (IWGSC, 2018), Gramene (http://www.gramene.org/), BLAST non-reduntant protein sequence (https://blast.ncbi.nlm.nih.gov/Blast.cgi?PROGRAM=blastx&PAGE_TYPE=BlastSearch&LINK_LOC=blasthome), and Taxonomy Browser (https://www.ncbi.nlm.nih.gov/Taxonomy/Browser/wwwtax.cgi?id=1437183). Source data are provided with this paper.

## Source data

Source data

## Code availability

The scripts used in these analyses have been published in GitHub [https://github.com/steuernb/GBS_introgression_line_analysis] and linked with Zenodo^115^.
