# Supplementary material for: Aegilops sharonensis genome-assisted identification of stem rust resistance gene Sr62
Source: Nat Commun. 2022 Mar 25;13:1607. doi: 10.1038/s41467-022-29132-8 (PMC8956640; doi:10.1038/s41467-022-29132-8)
Supplement: Supplementary file 1 — Supplementary Information [file 41467_2022_29132_MOESM1_ESM.pdf]

Aegilops sharonensis genome-assisted identification of stem rust resistance gene *Sr62*

Yu *et al.*

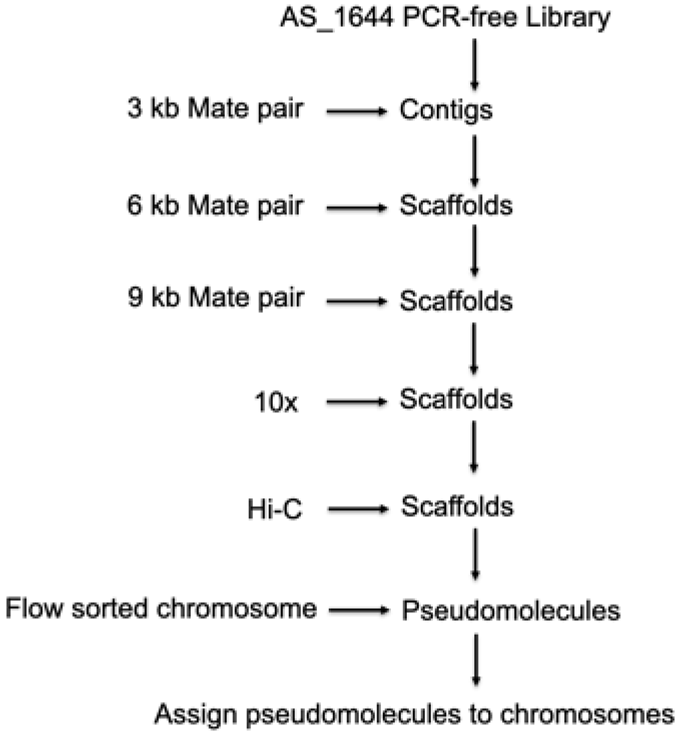

Supplementary Fig. 1. Flow chart depicting the strategy for sequencing and assembly of *Aegilops sharonensis* accession 1644 (AS_1644).

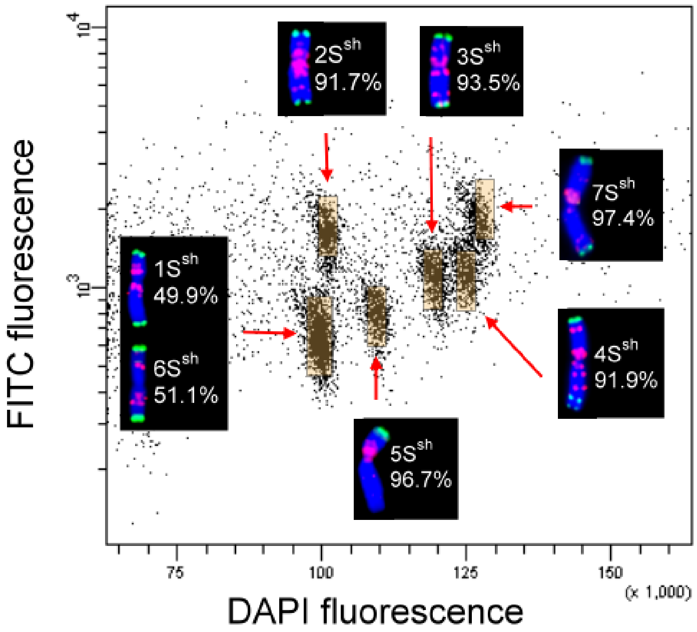

Supplementary Fig. 2. Flow karyotyping and chromosome sorting in *Ae. sharonensis*. The analysis of chromosomes labeled by FISHIS with FITC-labelled probes for GAA and ACG microsatellites and stained by DAPI resolved populations representing chromosomes 2S^{sh}, 3S^{sh}, 4S^{sh}, 5S^{sh} and 7S^{sh}, which could be flow-sorted at purities of 91 - 97%. Chromosomes 1S^{sh} and 6S^{sh} formed a composite population and were sorted simultaneously at approximately equal proportions. Insets: Chromosomes were assigned to the populations on flow karyotypes after FISH with probes for pSc119.2 (green), GAA$_n$ (red) and 45S rDNA (yellow); chromosomes were counterstained by DAPI (blue).

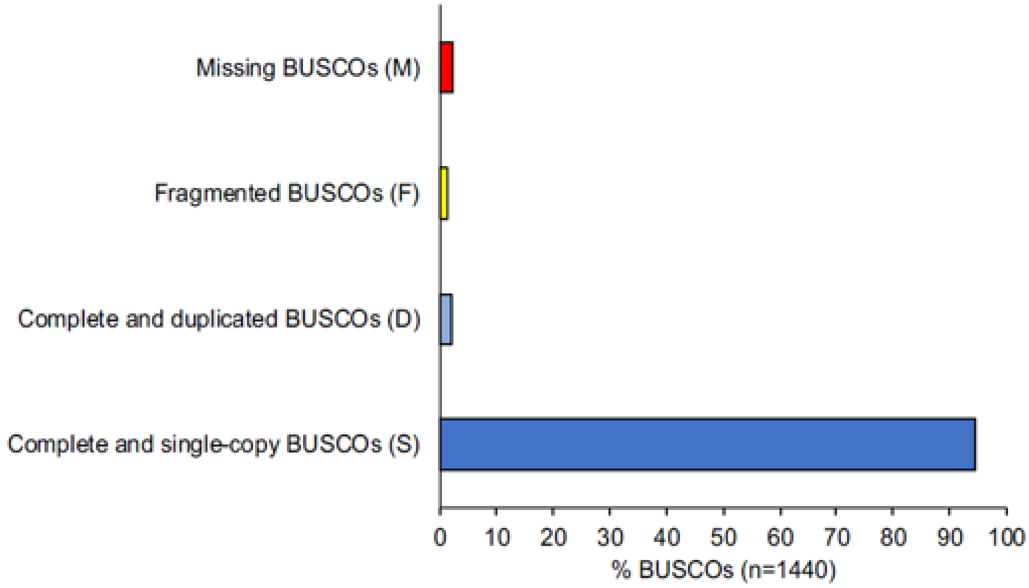

Supplementary Fig. 3. ***Aegilops sharonensis*** **accession 1644 genome BUSCO analysis.**

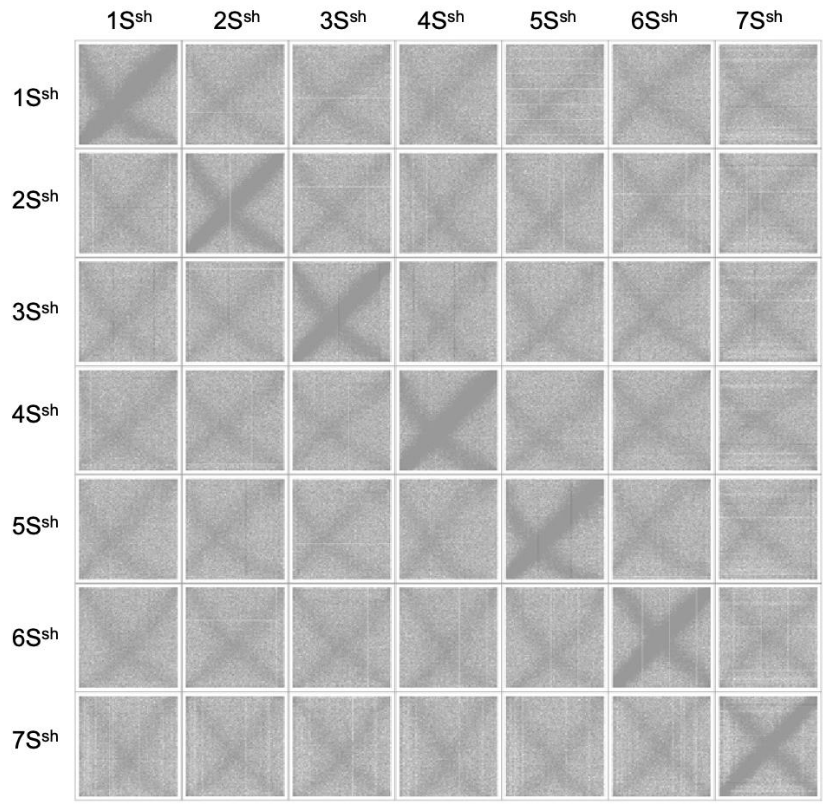

Supplementary Fig. 4. Inter-chromosome Hi-C contact matrices.

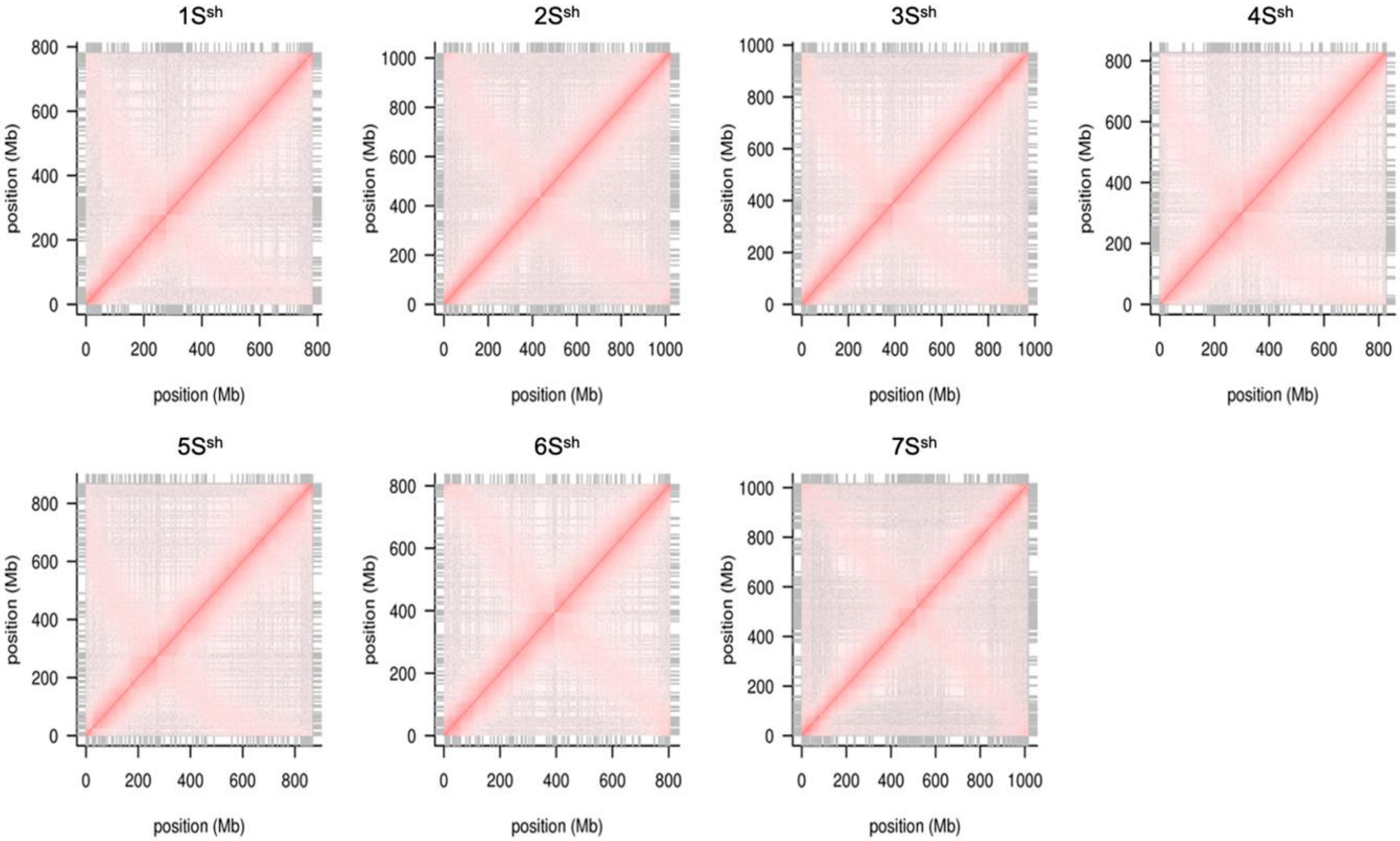

Supplementary Fig. 5. Intra-chromosome Hi-C contact matrices.

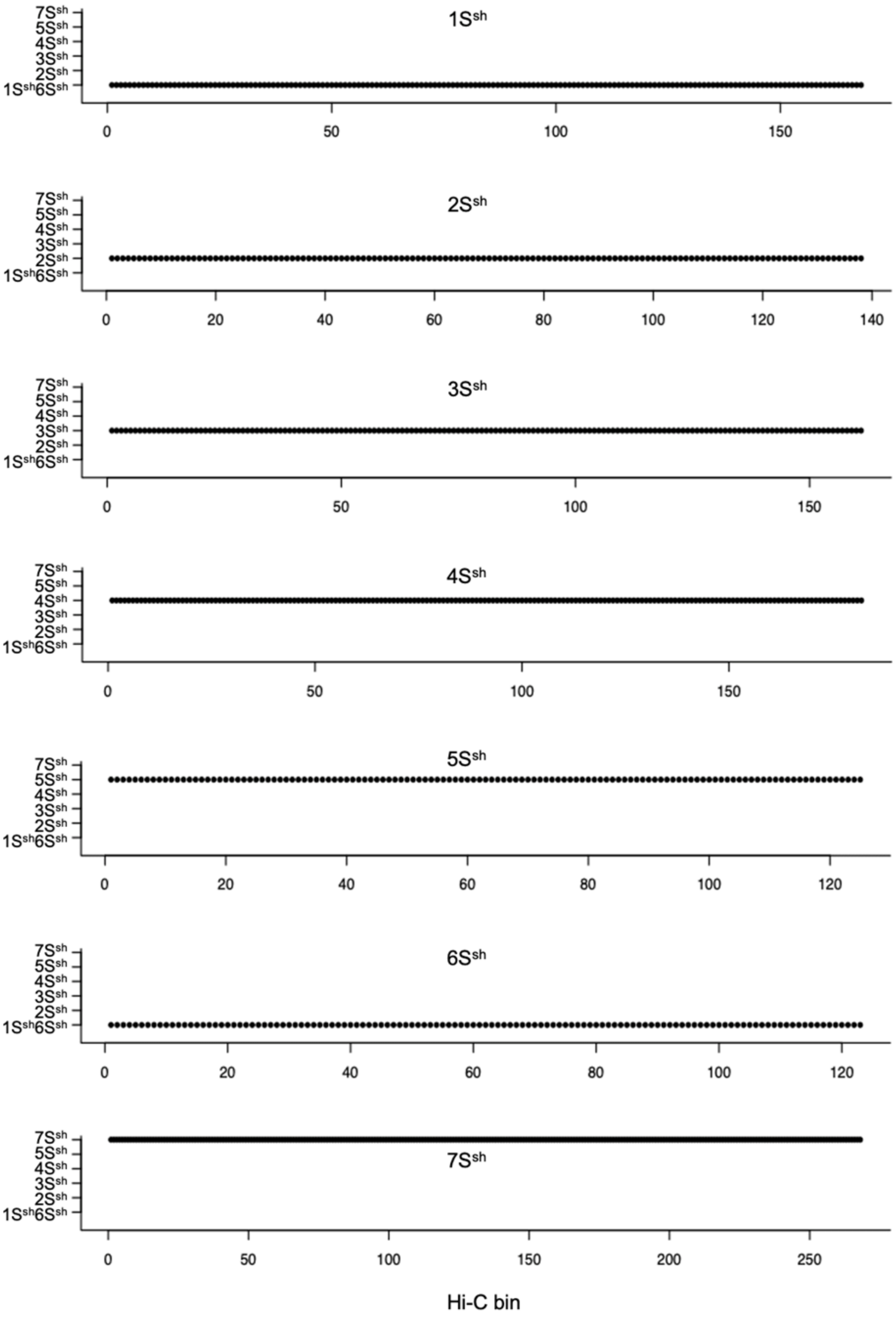

Supplementary Fig. 6. Concordance between Hi-C bin and chromosome flow sorting data.

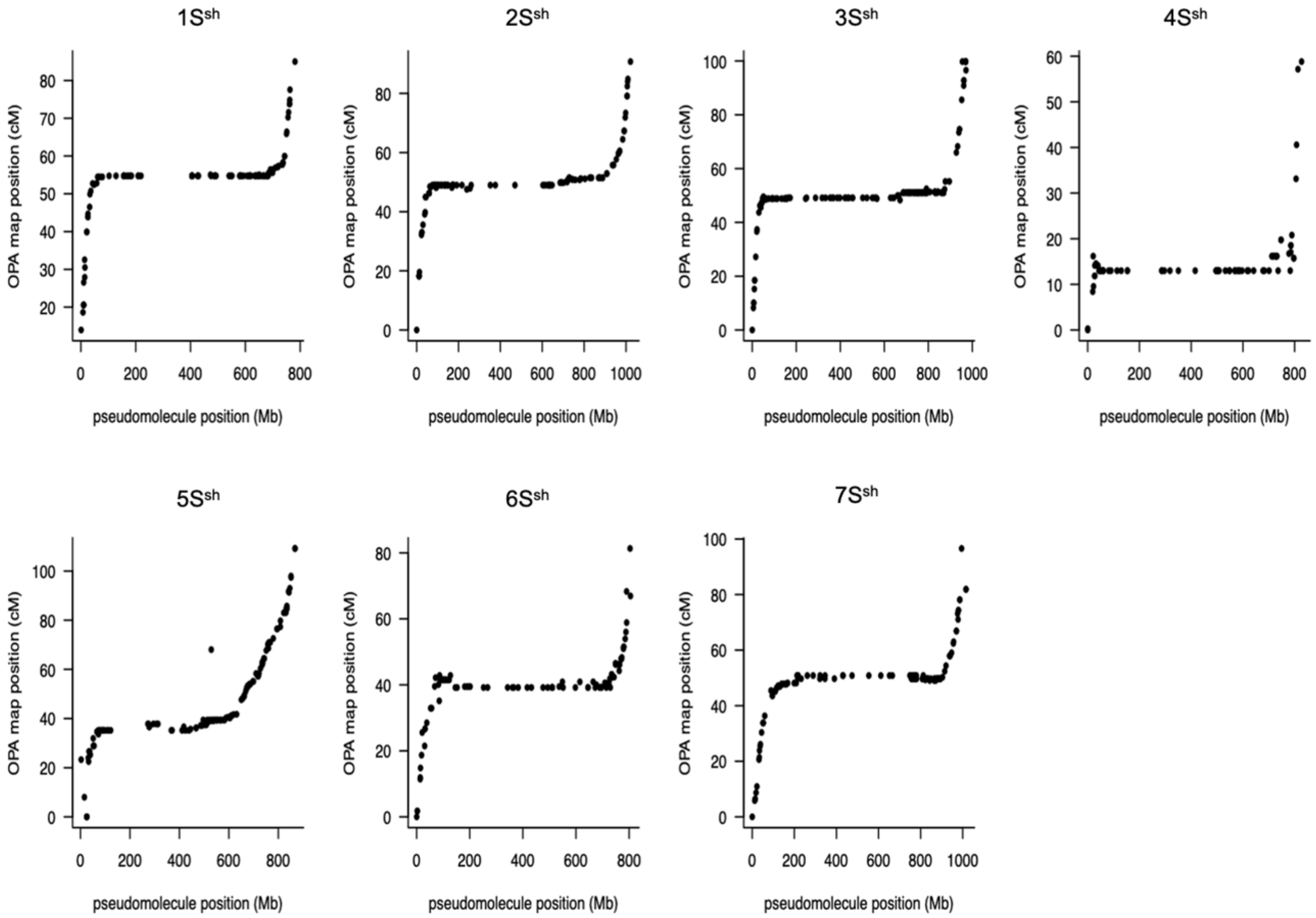

Supplementary Fig. 7. Comparison of the *Aegilops sharonensis* genetic map with the physical genome assembly.

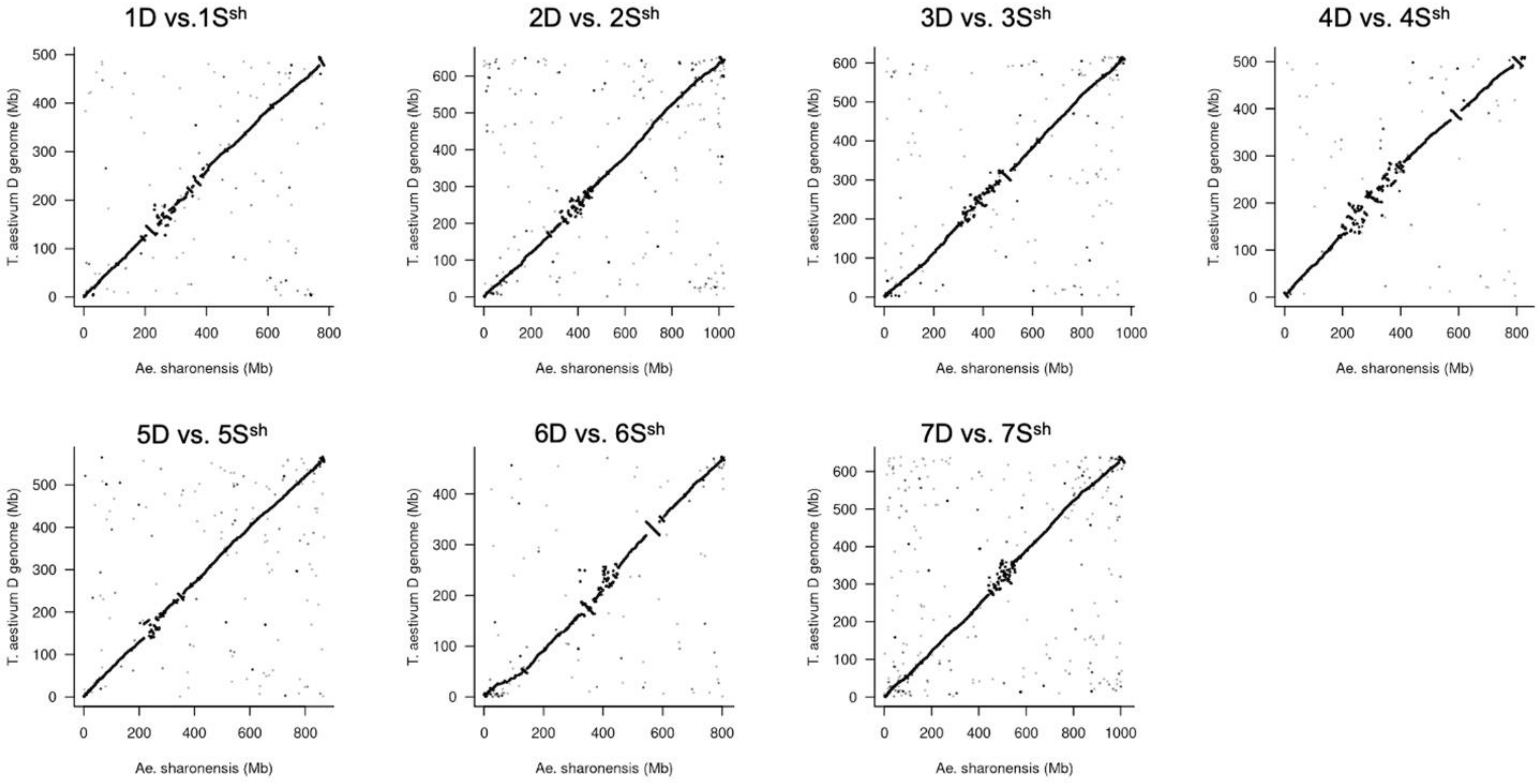

Supplementary Fig. 8. Comparison of the *Aegilops sharonensis* genome with the wheat cv. Chinese Spring D-subgenome.

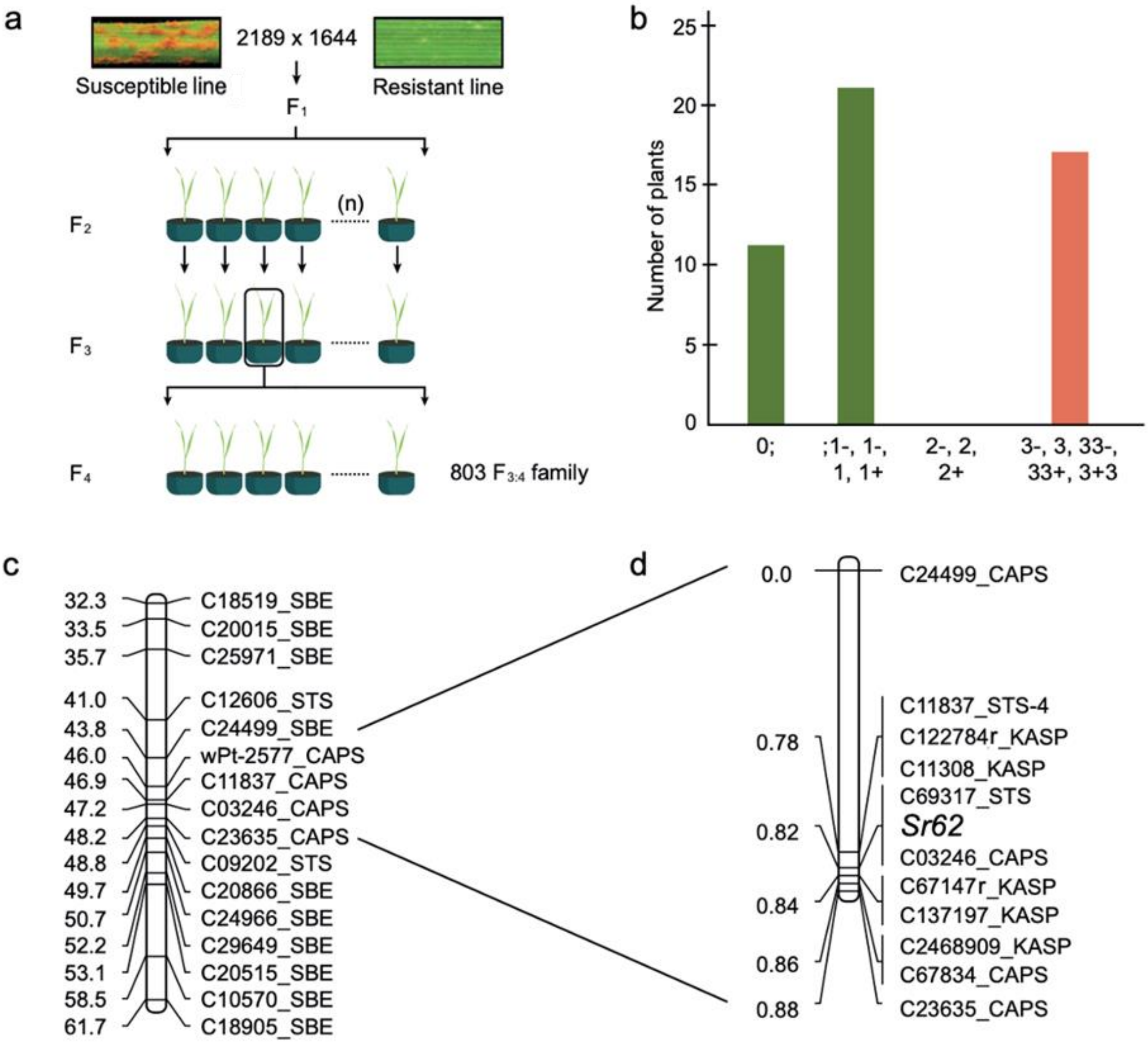

Supplementary Fig. 9. Genetic isolation of *Sr62* a. Development of the 803 family segregating for *Sr62*. The markers with SBE suffix are Sequenom markers. **b**. Infection type (IT) distribution in 49 progenies from the 803 family. **c**. Linkage map around *Sr62*. **d** Genetic position of *Sr62* in the linkage map.

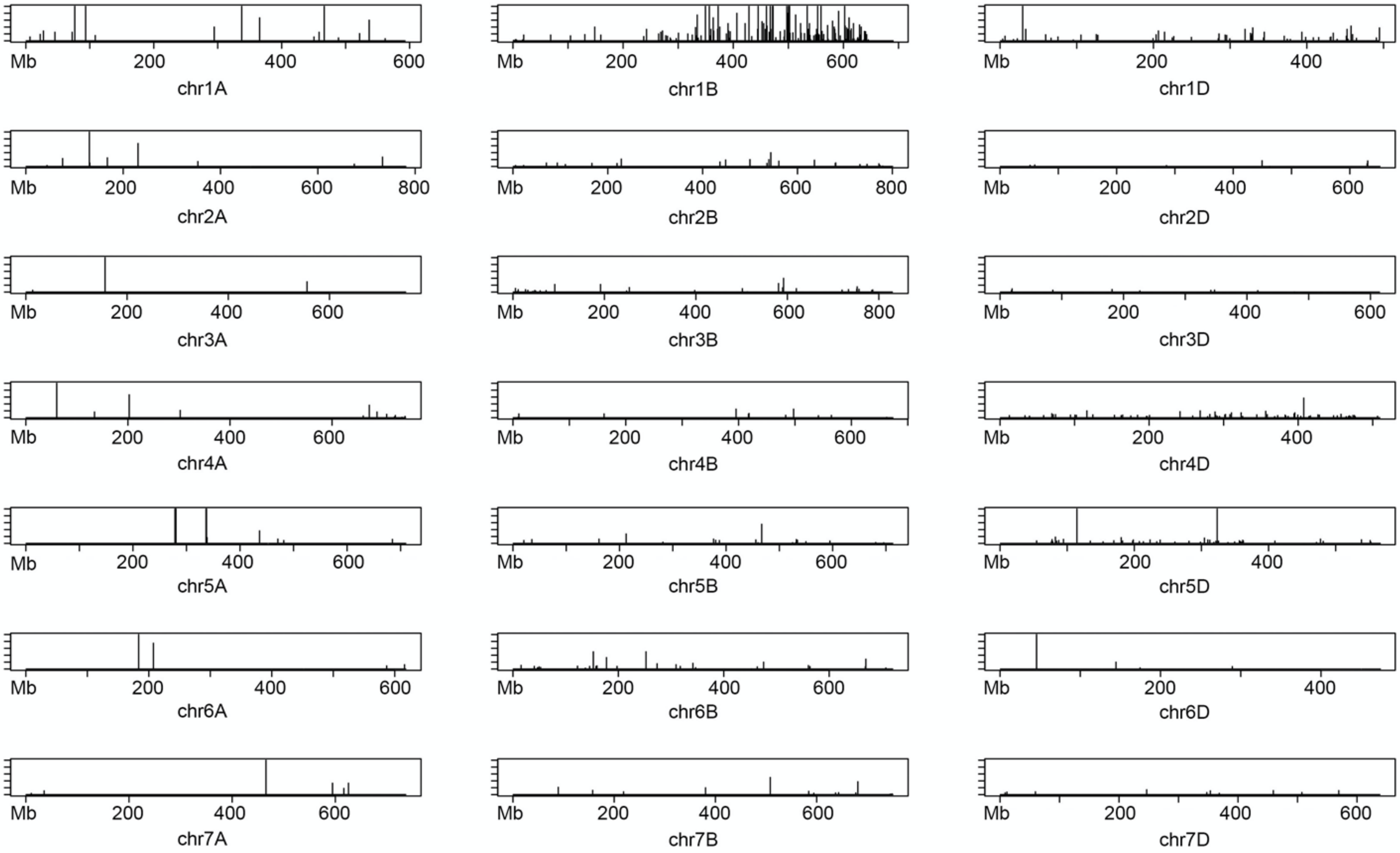

Supplementary Fig. 10. Plot of GBS on introgression line Zahir-1644 wild type.

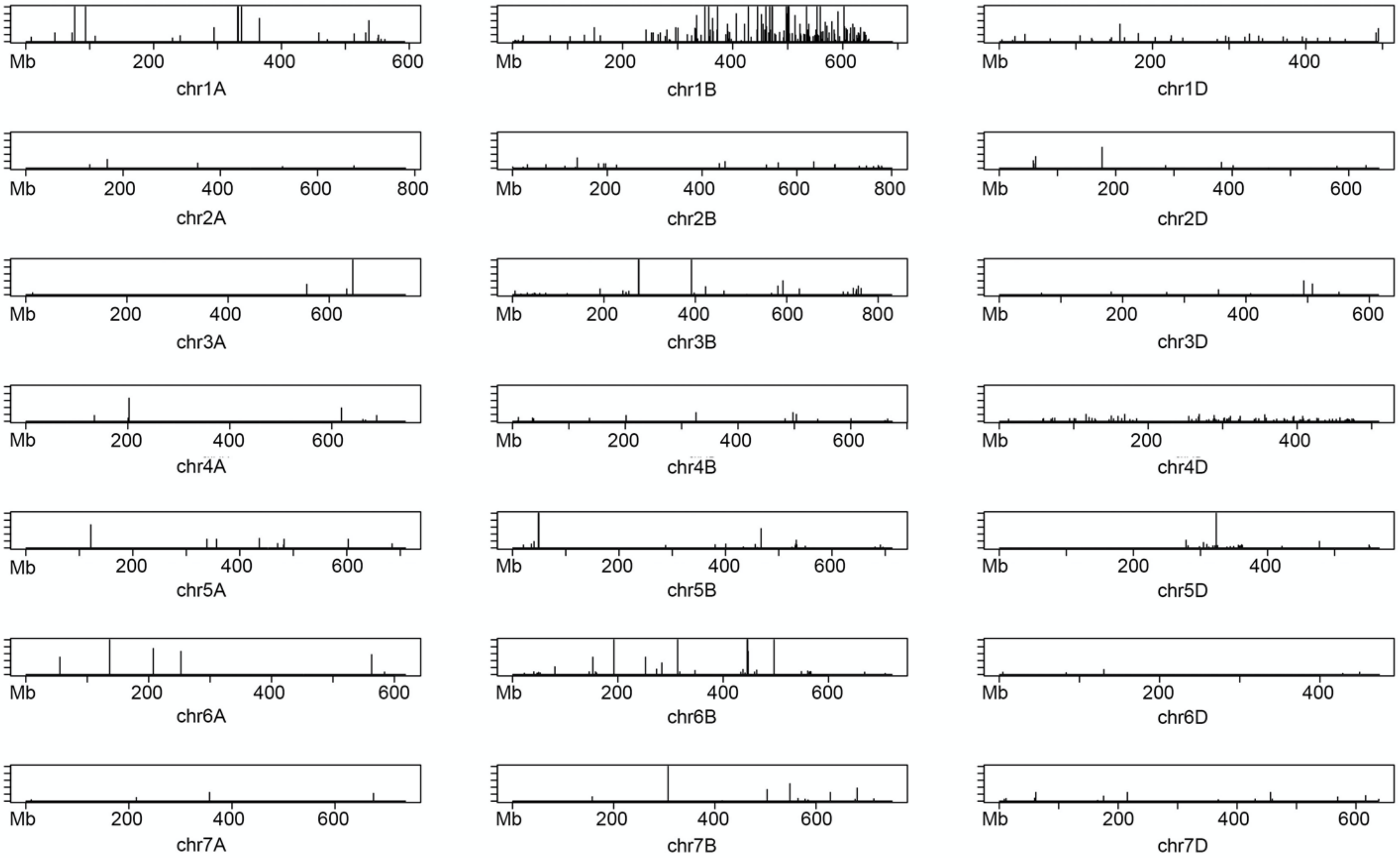

Supplementary Fig. 11. Plot of GBS on introgression line Zahir-1644 mutant 12a.

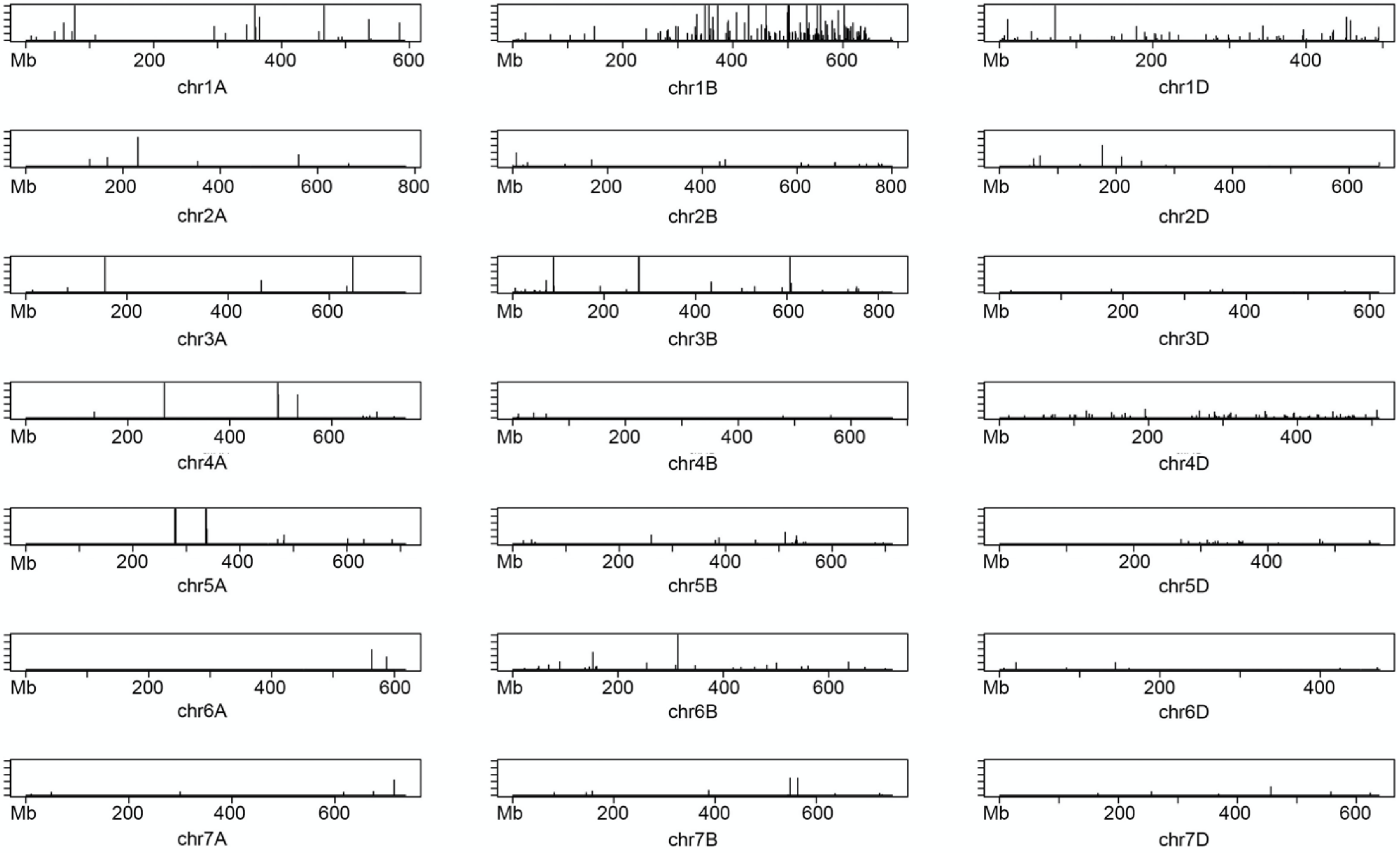

Supplementary Fig. 12. Plot of GBS on introgression line Zahir-1644 mutant 44d.

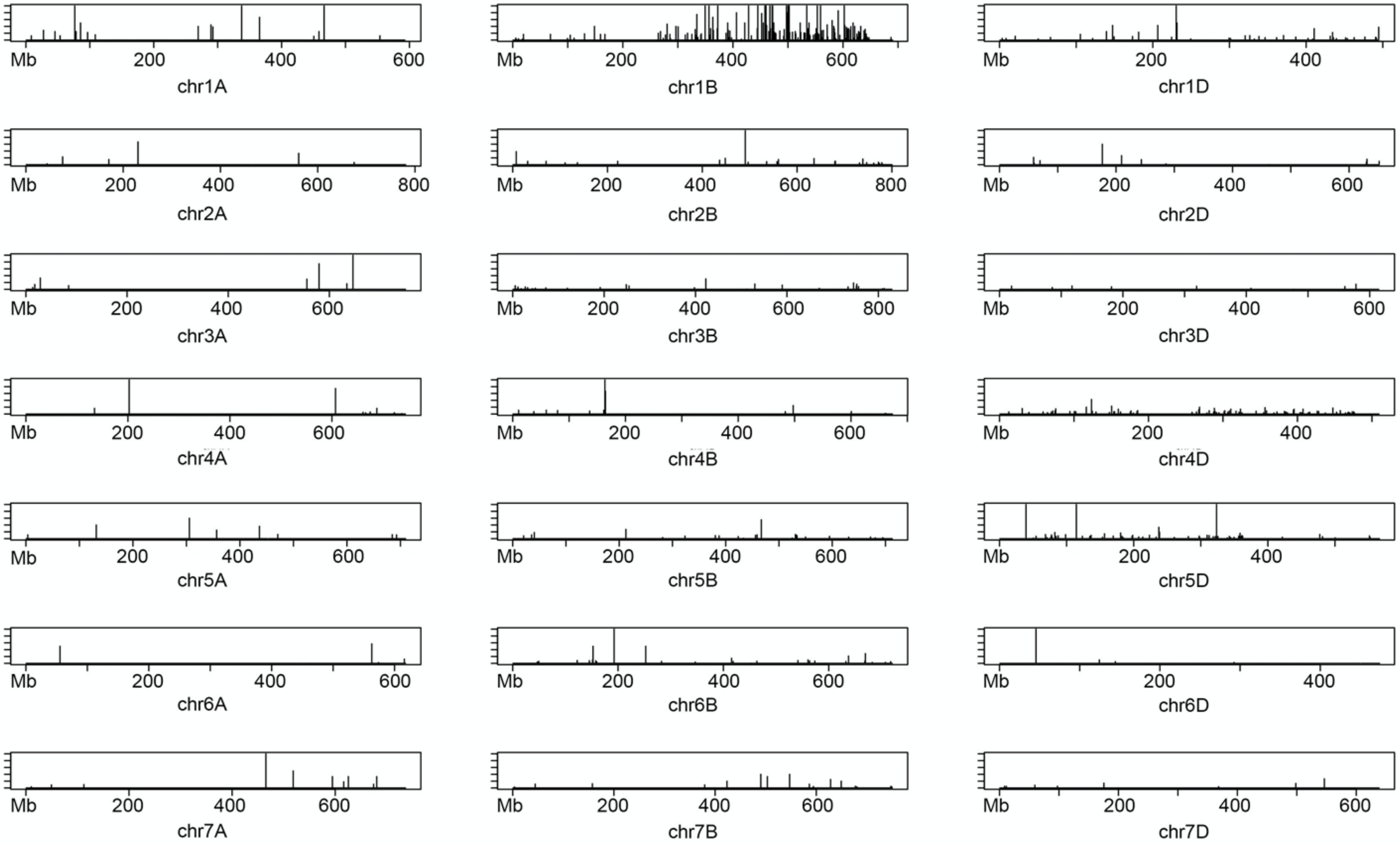

Supplementary Fig. 13. Plot of GBS on introgression line Zahir-1644 mutant 119d.

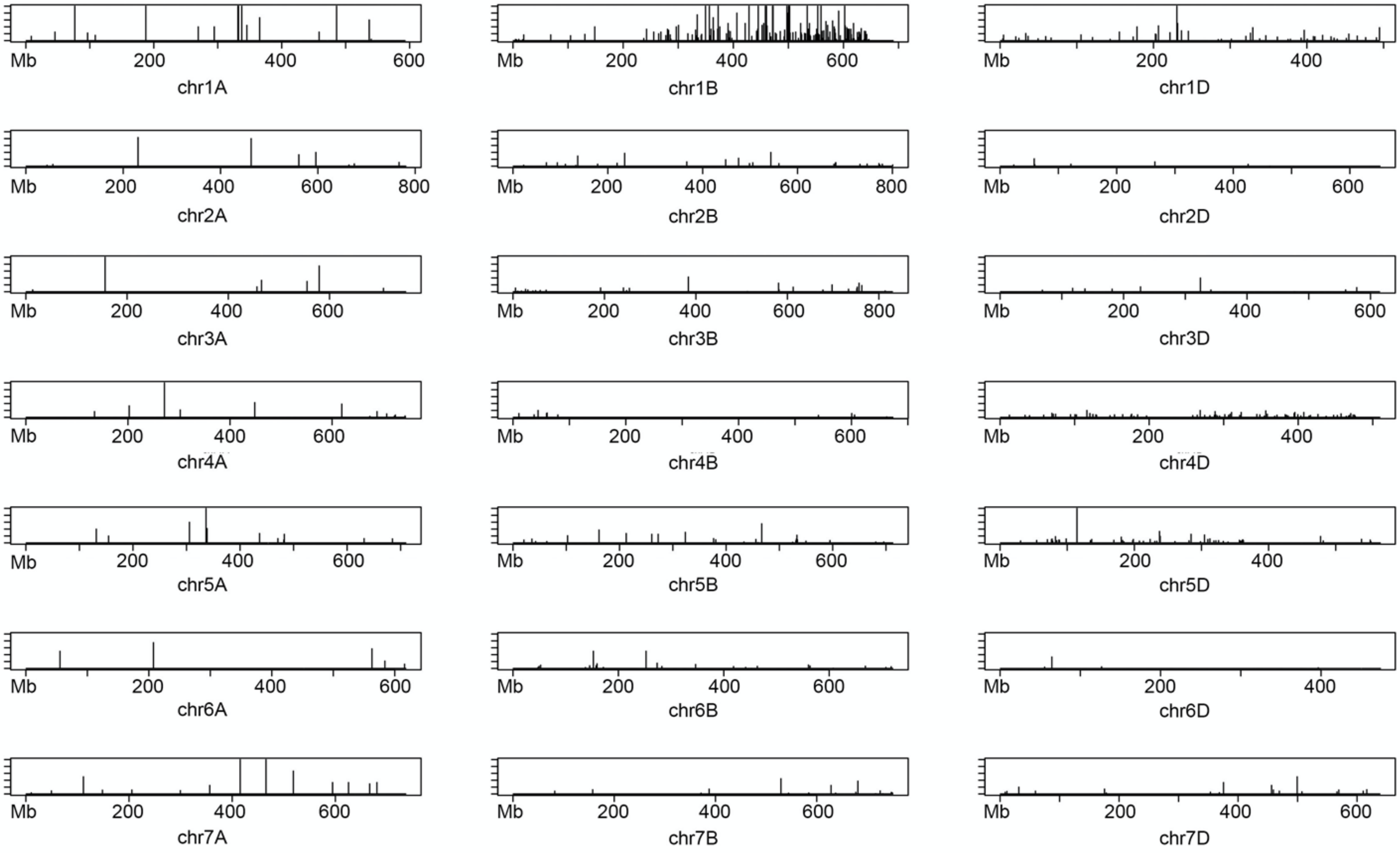

Supplementary Fig. 14. Plot of GBS on introgression line Zahir-1644 mutant 190d.

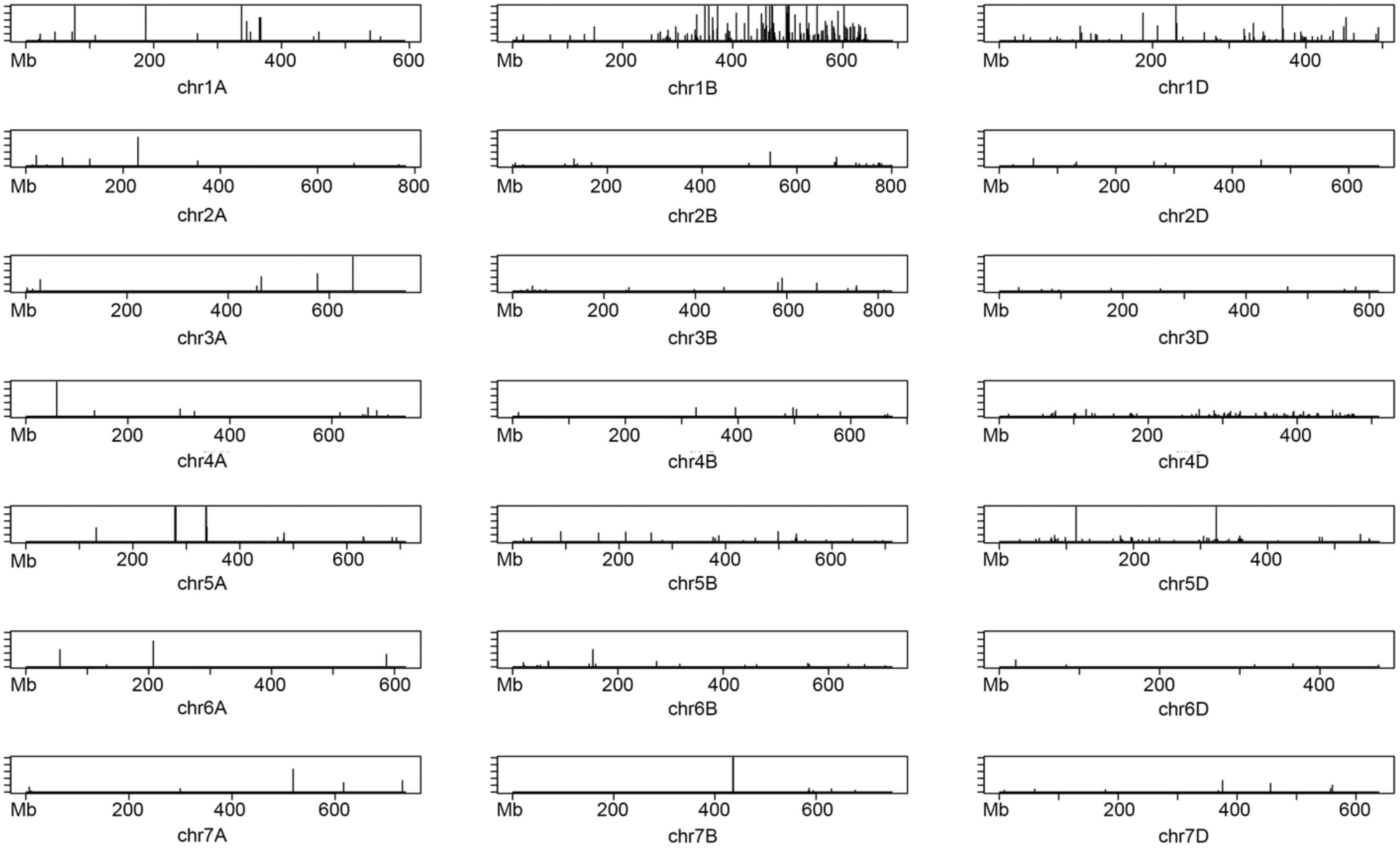

Supplementary Fig. 15. Plot of GBS on introgression line Zahir-1644 mutant 200h.

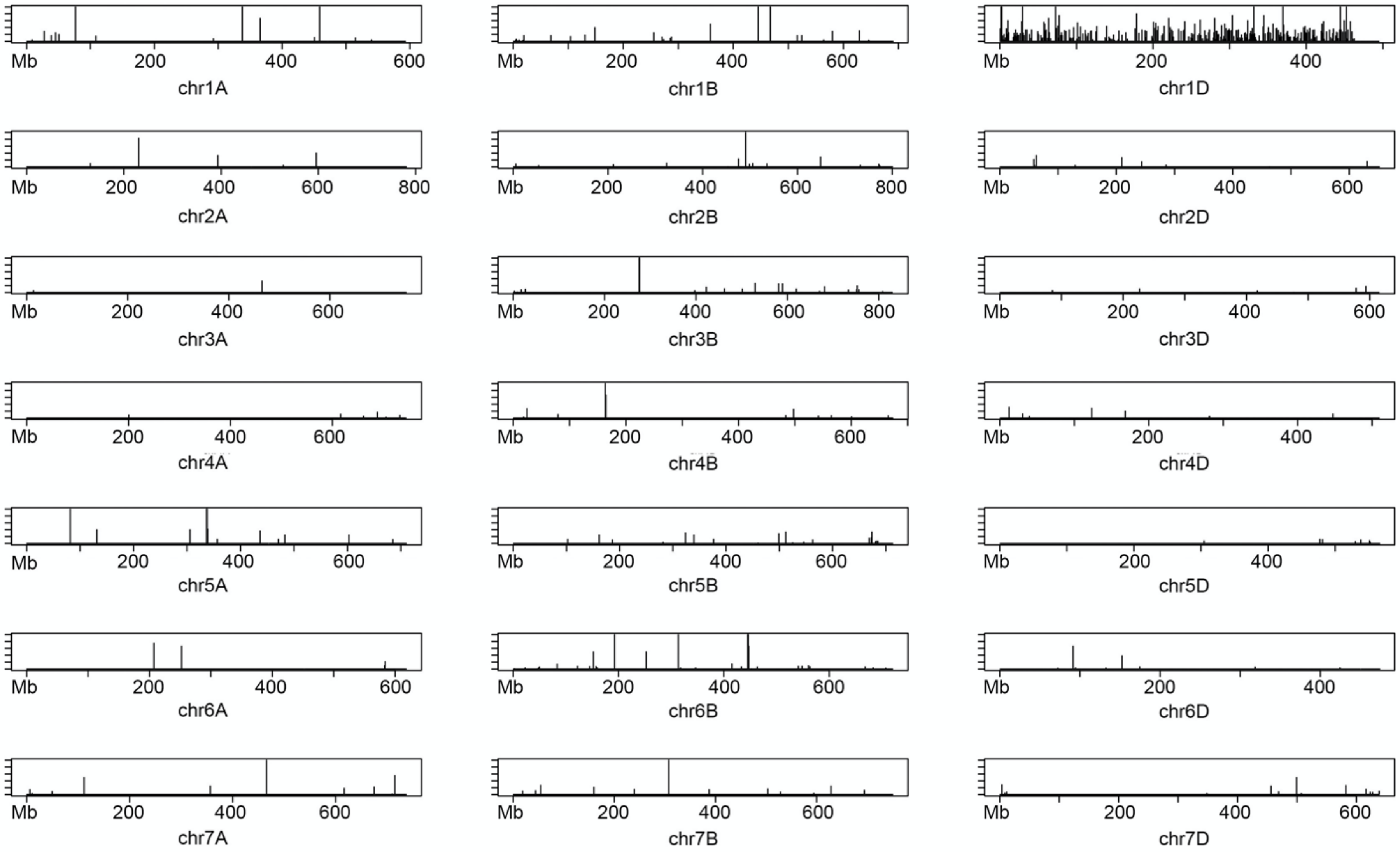

Supplementary Fig. 16. Plot of GBS on introgression line Zahir-1644 mutant 263g.

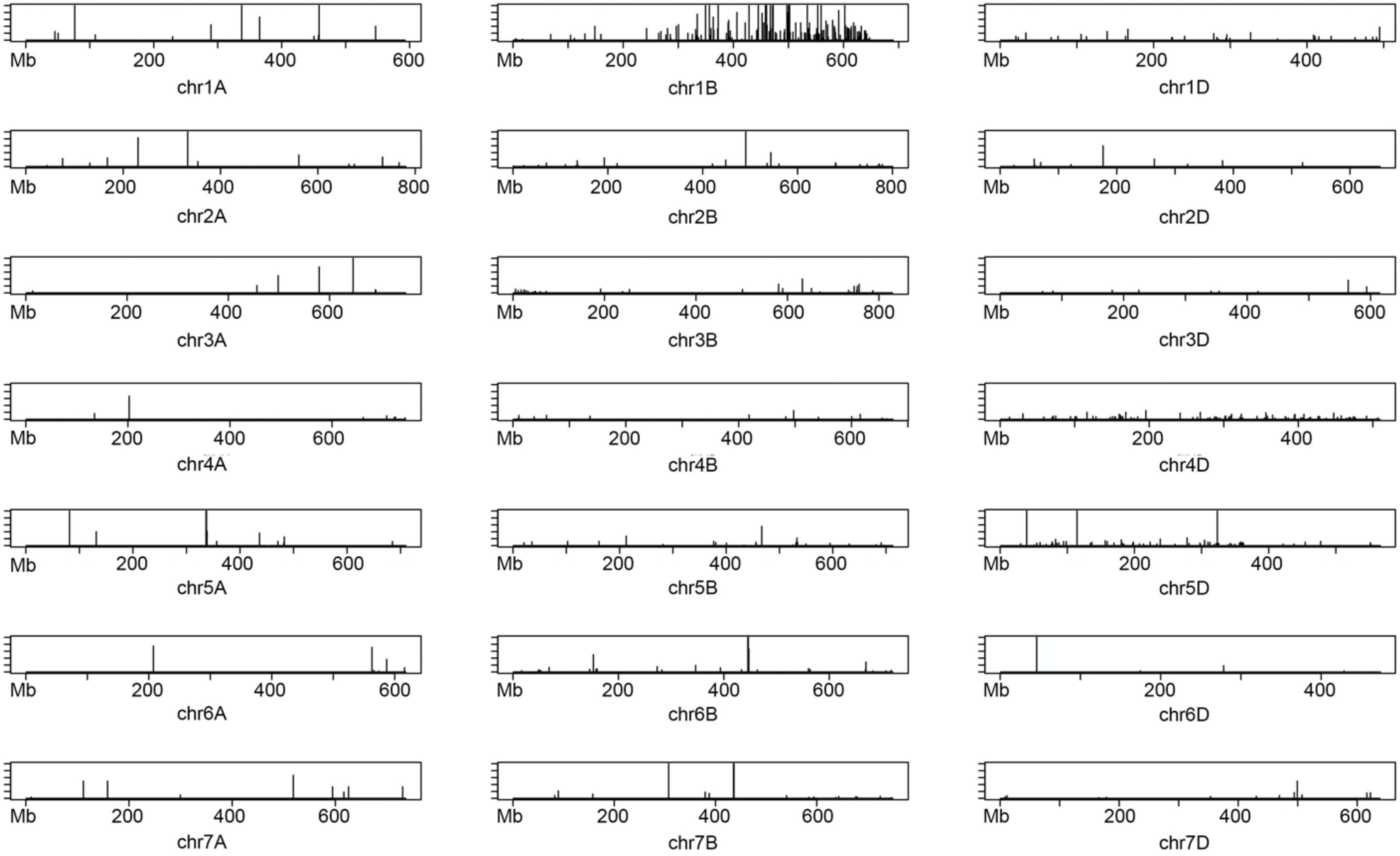

Supplementary Fig. 17. Plot of GBS on introgression line Zahir-1644 mutant 267d.

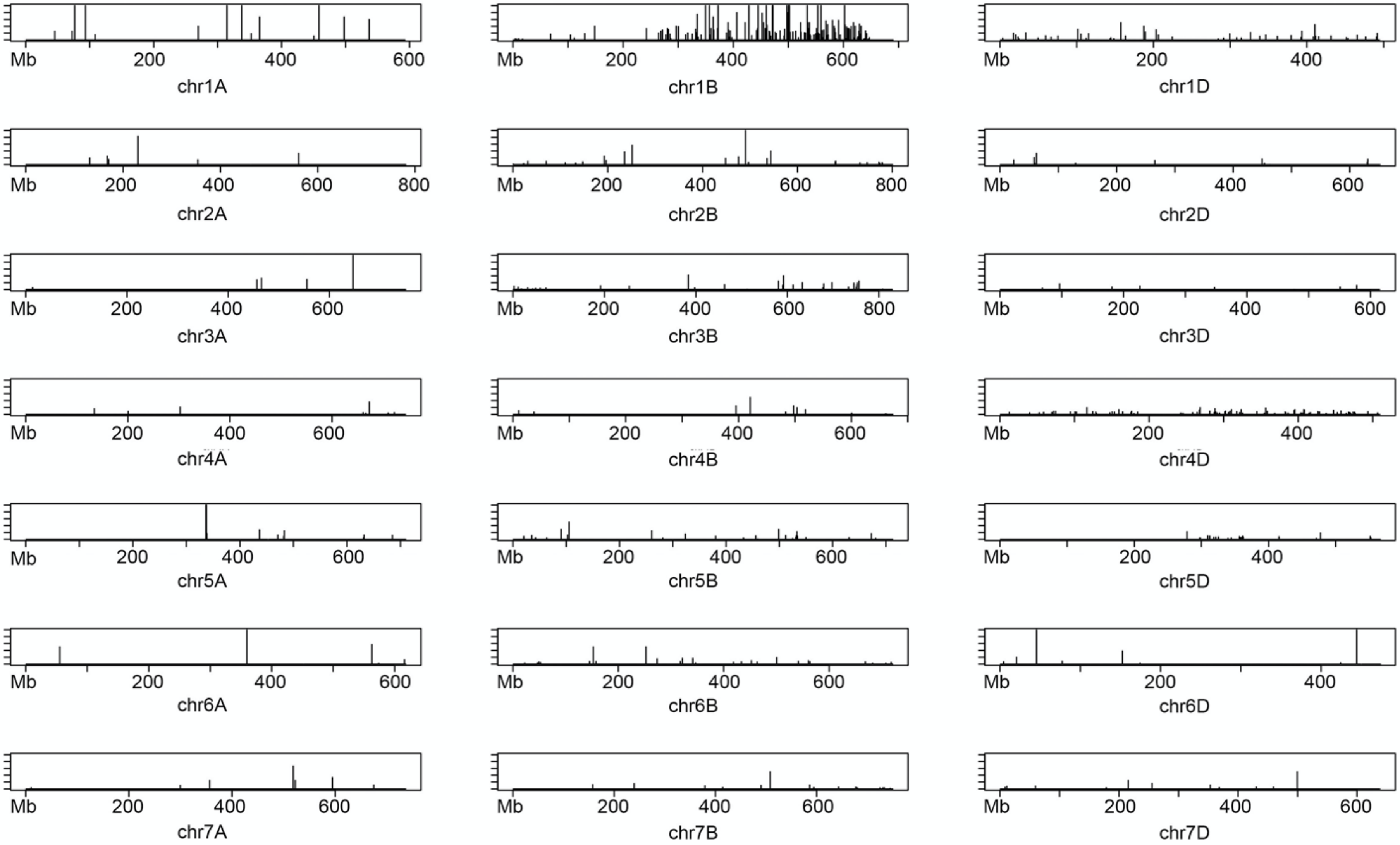

Supplementary Fig. 18. Plot of GBS on introgression line Zahir-1644 mutant 743a.

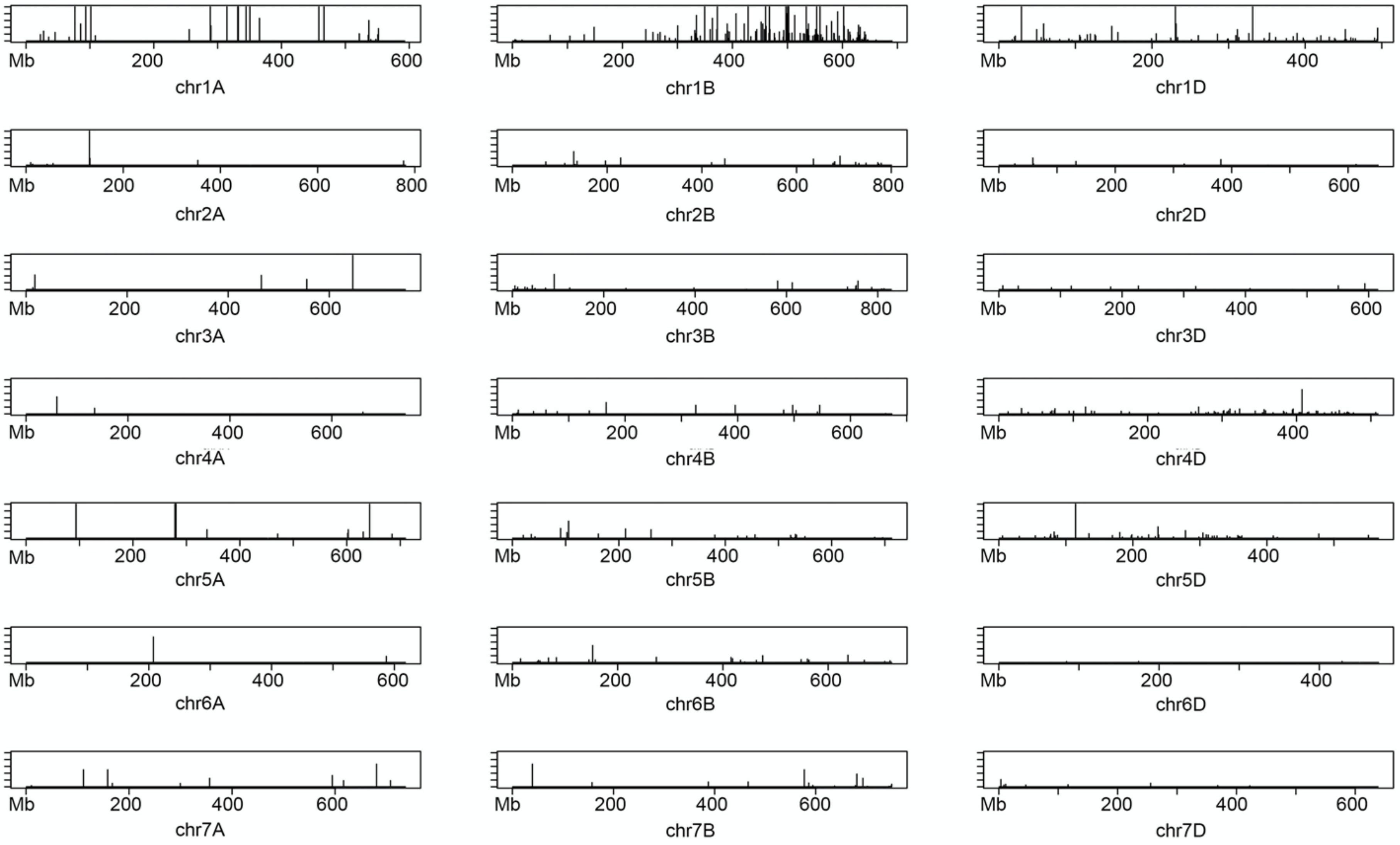

Supplementary Fig. 19. Plot of GBS on introgression line Zahir-1644 mutant 905c.

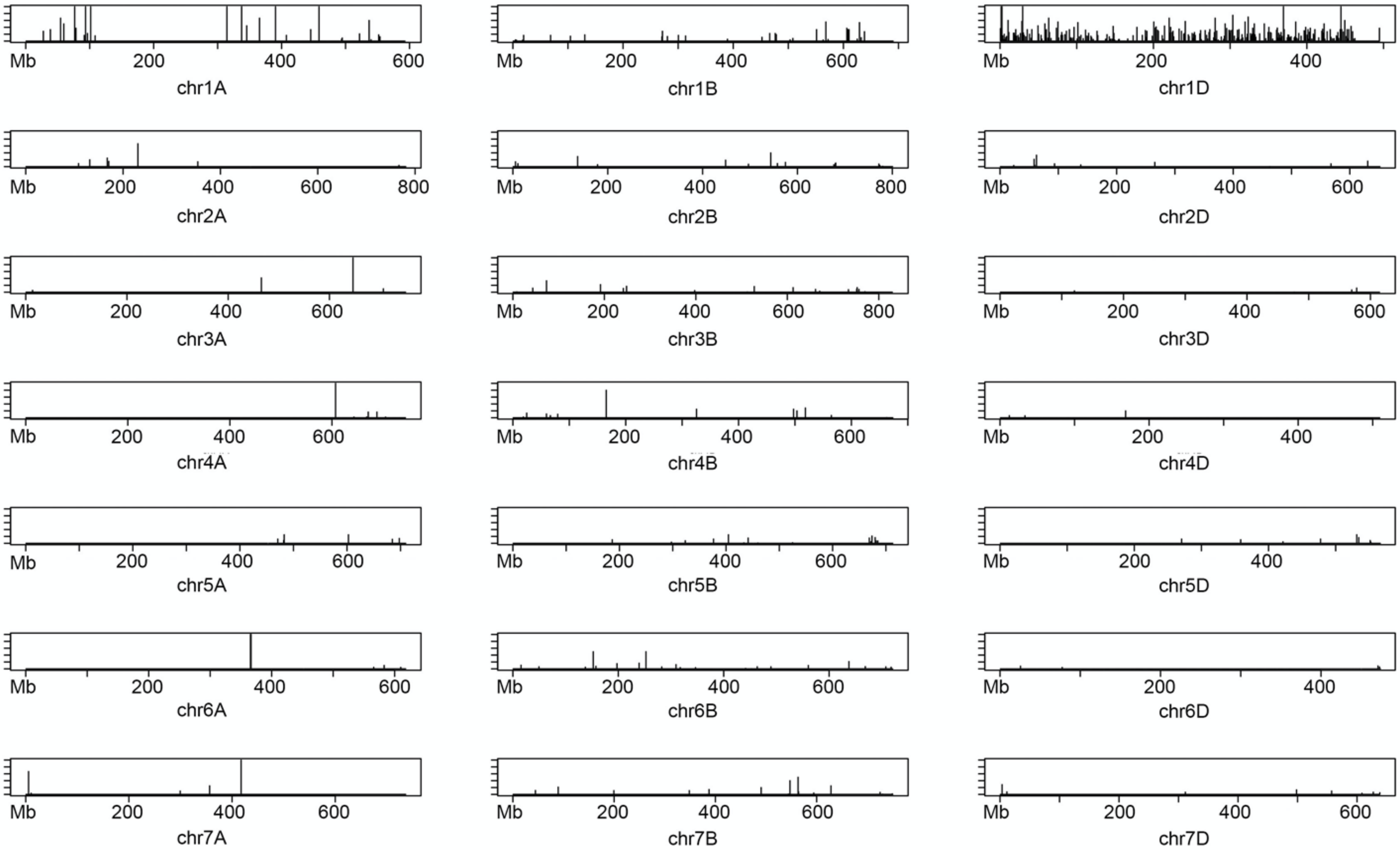

Supplementary Fig. 20. Plots of GBS on introgression line Zahir-1644 mutant 1298e.

a

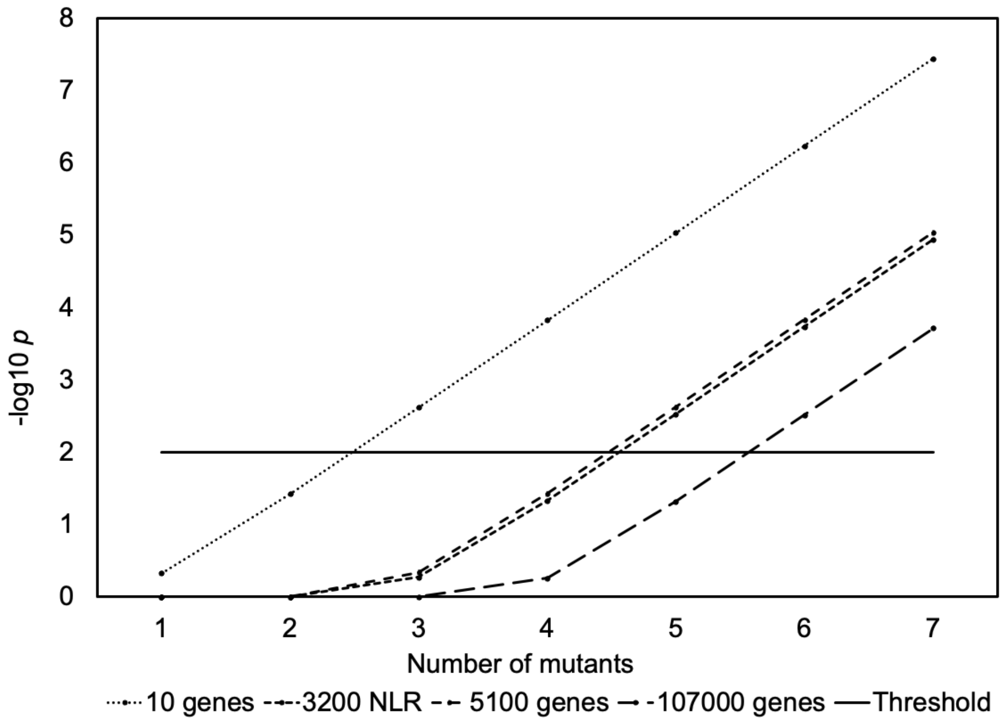

b

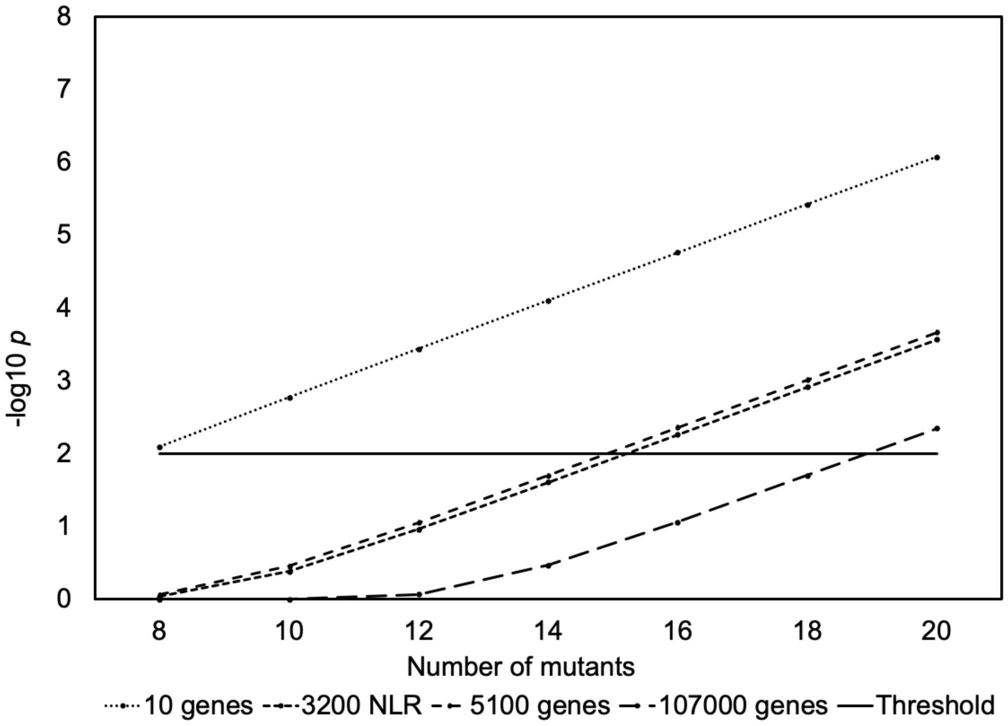

Supplementary Fig. 21. Probability of getting k out of n mutants with a SNV in the sequence of at least one of the genes investigated such as 10 genes in a fine mapping interval, 3200 NLR-encoding genes, all the 5100 genes of a chromosome, or all the 107000 genes in a whole genome of hexaploid wheat. a. Phenotype is controlled by one gene. **b**. Phenotype is controlled by two complementary genes.

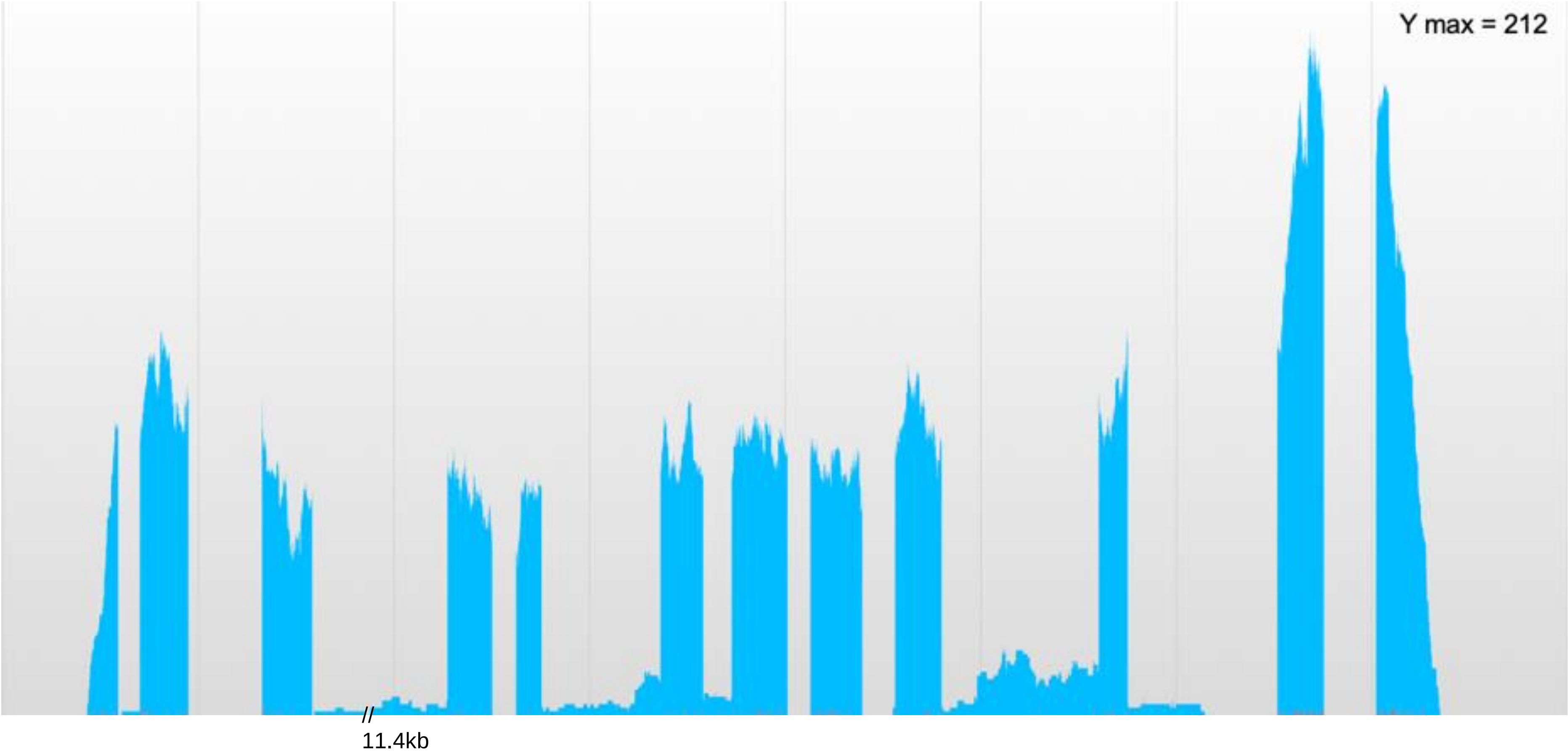

Supplementary Fig. 22. Mapping of RNA-Seq reads from the leaf transcriptome onto the *Sr62* construct insert (with the largest intron 11.4 kb removed).

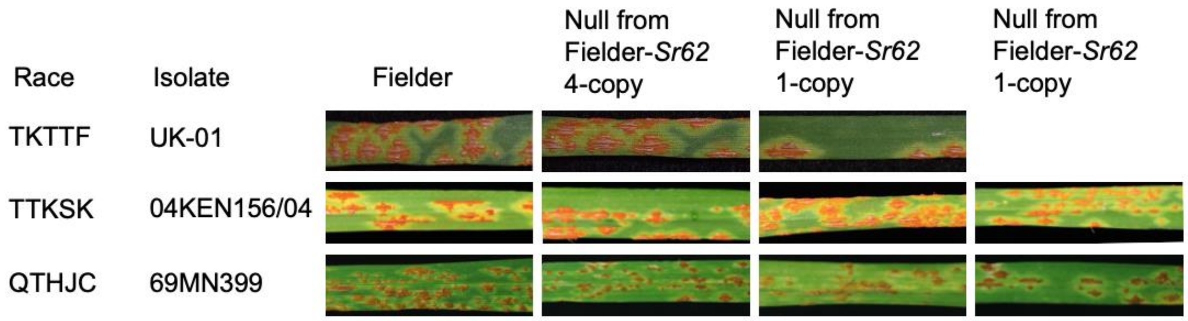

Supplementary Fig. 23. Reactions of T_1 null plants derived from three independent transgenic lines to three isolates of *Pgt*.

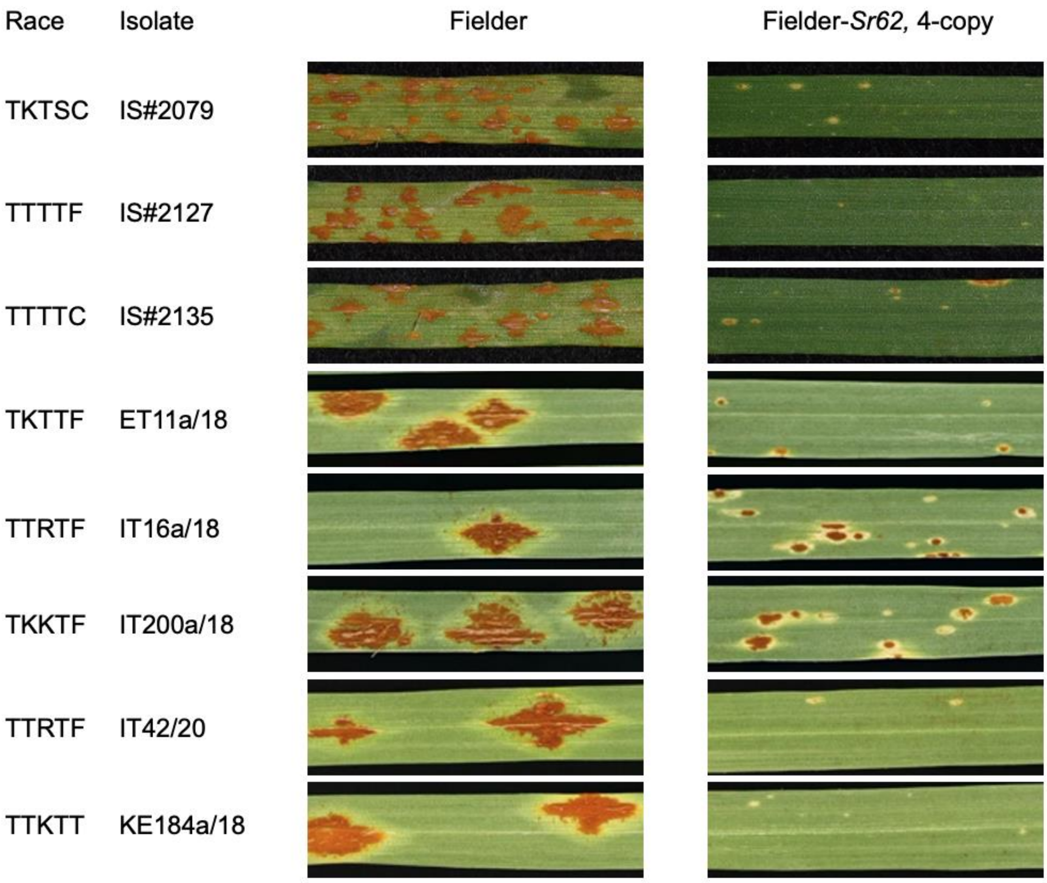

Supplementary Fig. 24. Reactions of T_1 plants derived from the 4-copy T_0 plant to an additional eight isolates of stem rust.

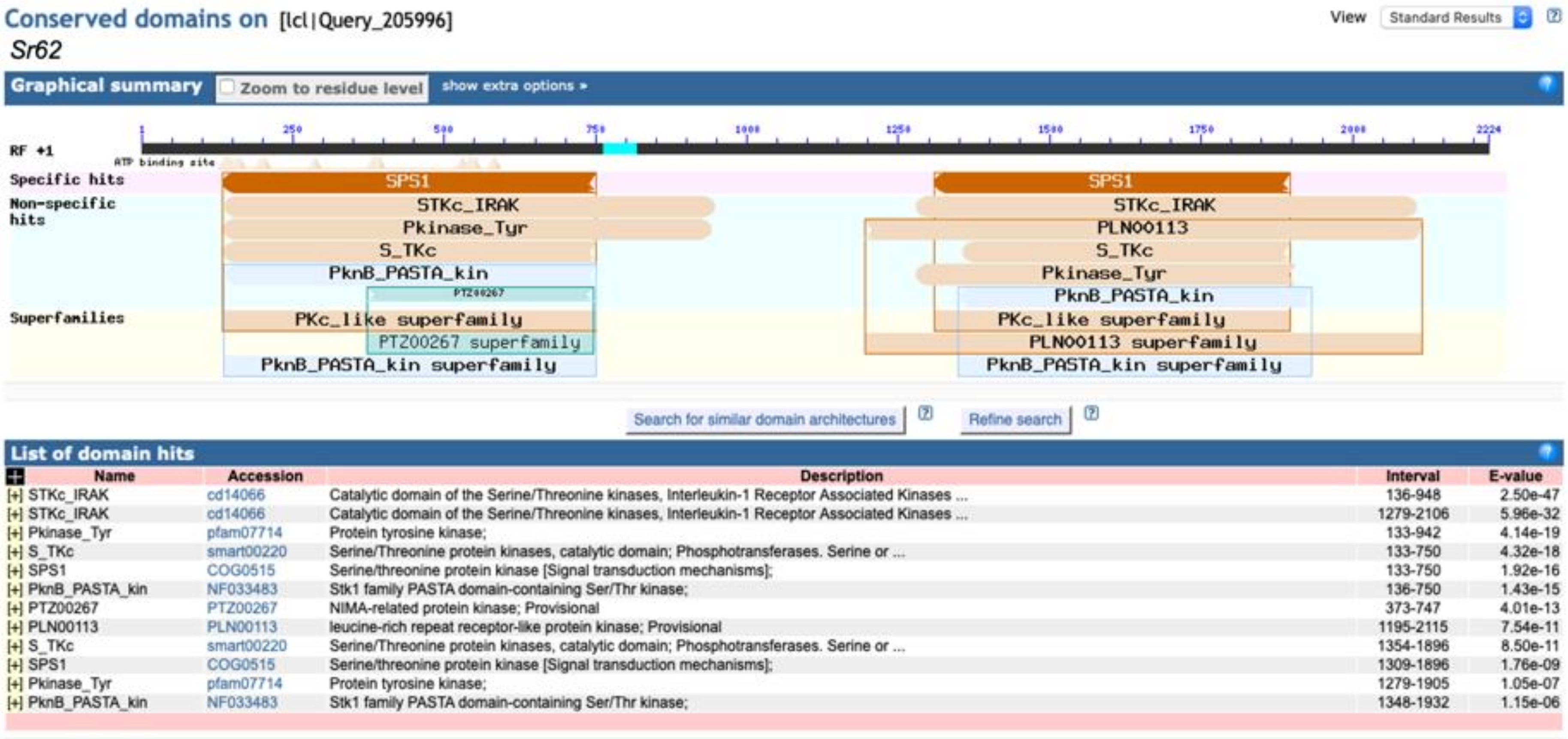

Name	Accession	Description	Interval	E-value
[+] STKc_IRAK	cd14066	Catalytic domain of the Serine/Threonine kinases, Interleukin-1 Receptor Associated Kinases ...	136-948	2.50e-47
[+] STKc_IRAK	cd14066	Catalytic domain of the Serine/Threonine kinases, Interleukin-1 Receptor Associated Kinases ...	1279-2106	5.96e-32
[+] Pkinase_Tyr	pfam07714	Protein tyrosine kinase;	133-942	4.14e-19
[+] S_TKc	smart00220	Serine/Threonine protein kinases, catalytic domain; Phosphotransferases. Serine or ...	133-750	4.32e-18
[+] SPS1	COG0515	Serine/threonine protein kinase [Signal transduction mechanisms];	133-750	1.92e-16
[+] PknB_PASTA_kin	NF033483	Stk1 family PASTA domain-containing Ser/Thr kinase;	136-750	1.43e-15
[+] PTZ00267	PTZ00267	NIMA-related protein kinase; Provisional	373-747	4.01e-13
[+] PLN00113	PLN00113	leucine-rich repeat receptor-like protein kinase; Provisional	1195-2115	7.54e-11
[+] S_TKc	smart00220	Serine/Threonine protein kinases, catalytic domain; Phosphotransferases. Serine or ...	1354-1896	8.50e-11
[+] SPS1	COG0515	Serine/threonine protein kinase [Signal transduction mechanisms];	1309-1896	1.76e-09
[+] Pkinase_Tyr	pfam07714	Protein tyrosine kinase;	1279-1905	1.05e-07
[+] PknB_PASTA_kin	NF033483	Stk1 family PASTA domain-containing Ser/Thr kinase;	1348-1932	1.15e-06

Supplementary Fig. 25. *Sr62* encodes a protein with two serine/threonine (S/T) kinase domains.

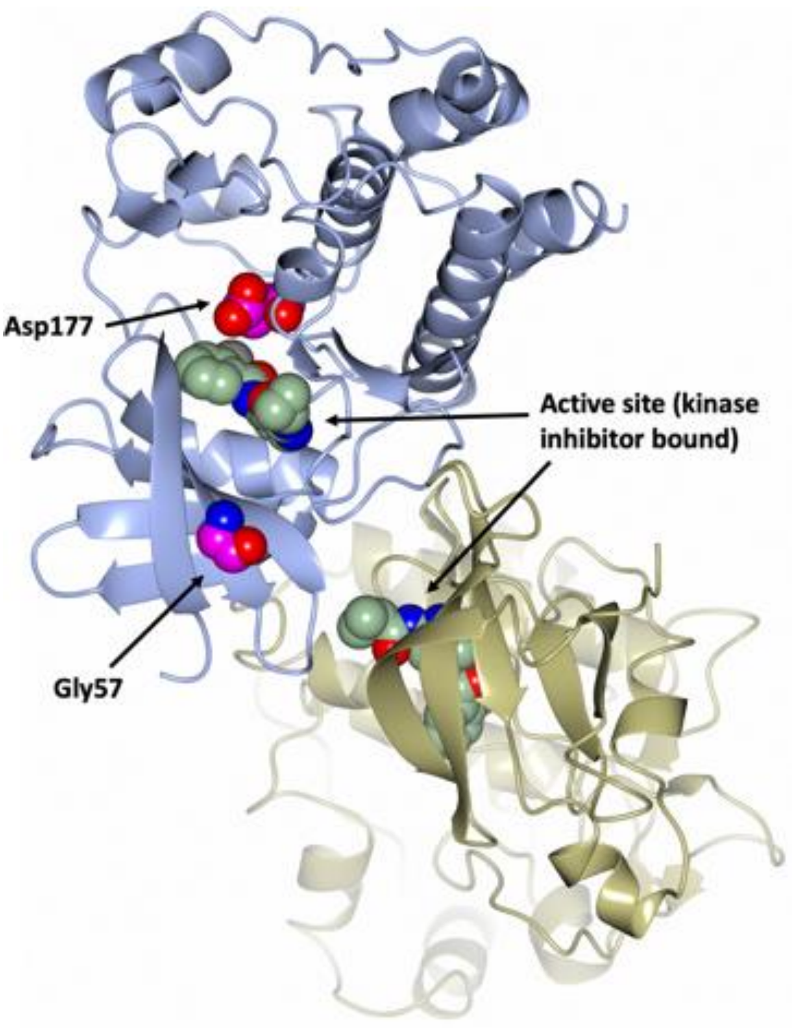

Supplementary Fig. 26. Homology model of *Sr62* generated by the Phyre2 server. The tandem kinase domains of *Sr62* were modelled on PDB entry 4OLI, the top template selected by Phyre2. The overall structure of the tandem kinase is shown in ribbon representation with the N-terminal kinase domain in blue and the C-terminal kinase domain in gold. The active site pockets in each domain are marked with the kinase inhibitor "compound 7012" (from PDB entry 4OLI) shown as spheres with carbon atoms in green. The locations of mutants 353g (Glycine57Arginine) and 12a (Aspartate177Asparagine) are shown as spheres of their wild-type residues with carbon atoms in magenta. Source data are provided as a Source Data file.

Supplementary Table 1. Libraries used in genome assembly of *Aegilops sharonensis* accession 1644.

**Library name**	**Shorthand**	**Sequencing chemistry**	**Output (Gb)**
Paired end 450	PE450	250 bp paired end	450.6
Paired end 800	PE800	150 bp paired end	295.4
3 kb mate pair	MP3	150 bp paired end	284.8
6 kb mate pair	MP6	150 bp paired end	283.0
9 kb mate pair	MP9	150 bp paired end	336.9
10X Chromium linked-reads	10X	150 bp paired end	250.0
Hi-C chromatin confirmation proximity	Hi-C	150 bp paired end	87.4
Flow sorted library chromosomes 1 and 6	Chrom_1_6	150 bp paired end	9.1
Flow sorted library chromosome 2	Chrom_2	150 bp paired end	8.6
Flow sorted library chromosome 3	Chrom_3	150 bp paired end	8.7
Flow sorted library chromosome 4	Chrom_4	150 bp paired end	8.7
Flow sorted library chromosome 5	Chrom_5	150 bp paired end	9.2
Flow sorted library chromosome 7	Chrom_7	150 bp paired end	10.0

Supplementary Table 2. The number of flow-sorted chromosomes, purity in the sorted fractions, and DNA yield after multiple displacement amplification.

Chromosome	Total no. of sorted chromosomes	Average purity in sorted fractions	DNA yield (two samples pooled)
$1S^{sh}$ + $6S^{sh}$	150 000	49.9% ($1S^{sh}$) + 51.1% ($6S^{sh}$)	8.45 µg
$2S^{sh}$	84 000	91.76%	8.17 µg
$3S^{sh}$	97 000	93.5%	8.42 µg
$4S^{sh}$	101 000	91.9%	8.43 µg
$5S^{sh}$	67 000	96.7%	8.53 µg
$7S^{sh}$	83 000	97.4%	8.31 µg

Supplementary Table 3. High confidence genes assigned to wheat cv. Chinese Spring chromosomes.

Chromosome	Genome	High confidence genes in Chinese Spring	Aligned*	%
1A	A	4344	3680	84.7
2A	A	5797	4912	84.7
3A	A	5235	4486	85.7
4A	A	4861	4092	84.2
5A	A	5429	4683	86.3
6A	A	4127	3467	84.0
7A	A	5552	4529	81.6
Total A-genome		35345	29849	84.5
1B	B	4714	4021	85.3
2B	B	6109	5232	85.6
3B	B	5905	5081	86.1
4B	B	3869	3434	88.8
5B	B	5576	4870	87.3
6B	B	4615	3868	83.8
7B	B	4855	4120	84.9
Total B-genome		35643	30626	85.9
1D	D	4484	3947	88.0
2D	D	5865	5169	88.1
3D	D	5318	4735	89.0
4D	D	3579	3310	92.5
5D	D	5563	4975	89.4
6D	D	4004	3458	86.4
7D	D	5399	4666	86.4
Total D-genome		34212	30260	88.4

* At 90% length, 90% identity.

Supplementary Table 4. Infection types (IT) and genotypes of the 803 family segregating for *Sr62*.

	Hv_1H_consensus_cM	U35_BRBH	Genomic distance in F2 (cM)	AS_01066	AS_01412	AS_00803	AS_00803-11	AS_00803-21	AS_00803-29	AS_00803-2	AS_00803-27	AS_00803-39	AS_00803-47	AS_00803-4	AS_00803-7	AS_00803-10	AS_00803-14	AS_00803-35	AS_00803-12	AS_00803-41	AS_00803-19	AS_00803-49	AS_00803-30	AS_00803-36	AS_00803-1	AS_00803-3	AS_00803-18	AS_00803-23	AS_00803-24	AS_00803-25	AS_00803-28	AS_00803-37	AS_00803-38	AS_00803-40	AS_00803-46	AS_00803-34	AS_00803-8	AS_00803-9	AS_00803-13	AS_00803-15	AS_00803-45	AS_00803-6	AS_00803-42	AS_00803-32	AS_00803-33	AS_00803-5	AS_00803-26	AS_00803-16	AS_00803-17	AS_00803-20	AS_00803-22	AS_00803-31	AS_00803-43	AS_00803-44	AS_00803-48
C25971_CAPS	15.7	U35_1078	35.7	b	a	a	a	a	a	a	a	a	a	a	a	a	a	a	a	a	a	a	a	a	a	a	a	a	a	a	a	a	a	a	a	a	a	a	a	a	a	a	a	a	a	a	a	a	a	a	a	a	a	a	a
C11627_CAPS	23.9	U35_2446		b	a	-	a	a	a	a	a	a	a	a	a	a	a	a	a	a	a	a	a	ab	ab	ab	ab	ab	ab	ab	ab	ab	ab	ab	ab	ab	ab	ab	ab	ab	ab	ab	ab	ab	ab	b	b	b	b	b	b	b	b	b	b
C24499_CAPS	26.1	U35_2603	43.8	b	a	ab	a	a	a	a	a	a	a	a	a	a	a	a	a	a	a	a	a	ab	ab	ab	ab	ab	ab	ab	ab	ab	ab	ab	ab	ab	ab	ab	ab	ab	ab	ab	ab	ab	ab	b	b	b	b	b	b	b	b	b	b
C07958_CAPS	33.6	U35_2055		b	a	-	a	a	a	a	a	a	a	a	a	a	a	a	a	a	a	a	a	ab	ab	ab	ab	ab	ab	ab	ab	ab	ab	ab	ab	ab	ab	ab	ab	ab	ab	ab	ab	ab	ab	b	b	b	b	b	b	b	b	b	b
C20569_CAPS	34.0	U35_3344		b	a	-	a	a	a	a	a	a	a	a	a	a	a	a	a	a	a	a	a	ab	ab	ab	ab	ab	ab	ab	ab	ab	ab	ab	ab	ab	ab	ab	ab	ab	ab	ab	ab	ab	ab	b	b	b	b	b	b	b	b	b	b
C07997_CAPS	34.8	U35_17207		b	a	-	a	a	a	a	a	a	a	a	a	a	a	a	a	a	a	a	a	ab	ab	ab	ab	ab	ab	ab	ab	ab	ab	ab	ab	ab	ab	ab	ab	ab	ab	ab	ab	ab	ab	b	b	b	b	b	b	b	b	b	b
C11837_CAPS	37.0	U35_3546		b	a	-	a	a	a	a	a	a	a	a	a	a	a	a	a	a	a	a	a	ab	ab	ab	ab	ab	ab	ab	ab	ab	ab	ab	ab	ab	ab	ab	ab	ab	ab	ab	ab	ab	ab	b	b	b	b	b	b	b	b	b	b
C03246_CAPS	37.0	U35_17276		b	a	-	a	a	a	a	a	a	a	a	a	a	a	a	a	a	a	a	a	ab	ab	ab	ab	ab	ab	ab	ab	ab	ab	ab	ab	ab	ab	ab	ab	ab	ab	ab	ab	ab	ab	b	b	b	b	b	b	b	b	b	b
C23635_CAPS	42.5	U35_16128	48.2	b	a	-	a	a	a	a	a	a	a	a	a	a	a	a	a	a	a	a	ab	ab	ab	ab	ab	ab	ab	ab	ab	ab	ab	ab	ab	ab	ab	ab	ab	ab	ab	ab	ab	ab	ab	b	b	b	b	b	b	b	b	b	b
C09202_CAPS	50.0	U35_1191		b	a	-	a	a	a	a	a	a	a	a	a	a	a	a	a	a	a	a	ab	ab	ab	ab	ab	ab	ab	ab	ab	ab	ab	ab	ab	ab	ab	ab	ab	ab	ab	ab	ab	ab	ab	ab	b	b	b	b	b	b	b	b	b
C20866_CAPS	67.0	U35_6932	49.7	b	a	ab	a	a	a	a	a	a	a	a	a	a	a	a	a	a	a	a	ab	ab	ab	ab	ab	ab	ab	ab	ab	ab	ab	ab	ab	ab	ab	ab	ab	ab	ab	ab	ab	ab	ab	ab	b	b	b	b	b	b	b	b	b
C24966_CAPS	88.2	U35_17780	50.7	b	a	ab	a	a	a	a	a	a	a	a	a	a	a	a	a	a	a	a	ab	ab	ab	ab	ab	ab	ab	ab	ab	ab	ab	ab	ab	ab	ab	ab	ab	ab	ab	ab	ab	ab	ab	ab	b	b	b	b	b	b	b	b	b
C29649_CAPS	96.6	U35_15915	52.2	b	a	ab	a	a	a	a	a	a	a	a	a	a	a	a	a	a	a	a	ab	ab	ab	ab	ab	ab	ab	ab	ab	ab	ab	ab	ab	ab	ab	ab	ab	ab	ab	a	a	ab	ab	ab	b	b	b	b	b	b	b	b	b
C20515_CAPS	106.0	U35_7542	53.1	b	a	ab	a	a	a	a	a	a	a	a	a	a	a	a	a	a	a	a	ab	ab	ab	ab	ab	ab	ab	ab	ab	ab	ab	ab	ab	ab	ab	ab	ab	ab	ab	a	a	ab	ab	ab	b	b	b	b	b	b	b	b	b
C10570_CAPS	3H	U35_14758	58.5	b	a	-	a	a	a	a	a	a	a	a	a	a	a	a	a	a	ab	ab	ab	ab	ab	ab	ab	ab	ab	ab	ab	ab	ab	ab	ab	ab	ab	ab	ab	ab	ab	a	a	b	ab	ab	ab	b	b	b	b	b	b	b	b
C18905_CAPS	118.0	U35_3021	61.7	b	a	-	a	a	a	a	a	a	a	a	a	a	a	a	a	a	ab	ab	ab	ab	ab	ab	ab	ab	ab	ab	ab	ab	ab	ab	ab	ab	ab	ab	ab	ab	ab	a	a	b	ab	ab	ab	b	b	b	b	b	b	b	b
IT to TTKSK				0;	34Z		33+	33+	33+	33-	33-	33-	33-	3+3	3+3	3+3	3+3	3-	3	3	33+	3-	33+	1+	1-	1-	1-	1-	1-	1-	1-	1-	1-	1-	1-	1	;1-	;1-	;1-	;1-	;1-	1-	1-	;1-	0;	0;	0;	0;	0;	0;	0;	0;	0;	0;	0;
Resistance				R	S	-	S	S	S	S/I	S	S/I	S/I	S	S	S	S	S/I	S	S	S	I	S	R	R	R	R	R	R	R	R	R	R	R	R	R	R	R	R	R	R	R	R	R	R	R	R	R	R	R	R	R	R	R	R

Genotyping marker on 2189 = a

Genotyping marker on 1644 = b

Heterozygote marker = ab

AS_01066 = 1644

AS_01412 = 2189

Supplementary Table 5. Primers of the CAPS markers used in this study.

Marker name	Type	Primer sequence 5'-3' (Forward)	Primer sequence 5'-3' (Reverse)	Enzyme	Annealing temperature
C25971_CAPS	CAPS	GACTCCTCTTCTTTCCCCTC	GCGGCGACTACTTGTTCTTG	*Ape*KI	56° C
C11627_CAPS	CAPS	AGAATAAGGCGAGGCTGCAT	GTTGCCCAAAAGGGTTACAA	*Tru1*I	56° C
C24499_CAPS	CAPS	AGCCAAAAGAAGAGAGTTGCTG	CAATGGCTATCTTGGGCAAA	*Bst*UI	55° C
C07958_CAPS	CAPS	CCCGTCCTCCTTATCTCCA	ATGGATTTCAGGGACAAGCA	AvrII	56° C
C20569_CAPS	CAPS	AGACCAACAGCACCAAGTCC	CACGTCGTCCCCATCTTC	*Bgl*I	56° C
C07997_CAPS	CAPS	CCCTACCCAGAAAATGATGC	CCTTTCCAGCTCAACCATTG	*Eco*RI	56° C
C11837_CAPS	CAPS	TAGGGCTCACCACCATCTTC	GGCTGAAGGAGTGGCATTT	*Kpn*I	56° C
C03246_CAPS	CAPS	TTGGGCTATTCGACAATGAA	AGAAGGGGGTATCGATGAGG	*Eco*RV	56° C
C23635_CAPS	CAPS	TTTGGTTGATGGTGGTAACG	ATGGAGTTCTCCCAGCAGAG	*Hpy*AV	56° C
C09202_CAPS	CAPS	CGTCTGCGTTTTCCTCGT	CGAACGATATGTTGGCCC	*Rsa*I	56° C
C20866_CAPS	CAPS	CCATAAATCCCCACTATGCAA	CATCGGGAGAACACTCTGGT	*Eco*RI	56° C
C24966_CAPS	CAPS	CCCTAACAACGGCAAGAG	AGCACCTGTAAAACTGAAC	*Drd*I	56° C
C29649_CAPS	CAPS	CCCTGGAAGTAAGATACATTACTCGT	GCCGAAGAAGGTAAAATTCCA	*Ban*I	56° C
C20515_CAPS	CAPS	GGAGTGCAAGCAACAGTGAG	ATGTAGCGCACACAAACACC	*Aci*I	56° C
C10570_CAPS	CAPS	GAGGTGCTCGACAACAGCAT	CTGTGGCTTGCTCAACAAAA	*Pst*I	56° C
C18905_CAPS	CAPS	CAACGTCAGGTCCATCCTCT	CGTGTGGACTGAGAACTTcG	*Eco*130I	56° C
C67834_CAPS	CAPS	ACCGGCCTAGCAAGGATTAT	CTCGACCATGGAGGATGAGT	*Bse*NI	56° C
wPt-2577_CAPS	CAPS	GCAGCCCTCCAGATCCTAGTTG	ATTCAAGGCACTGCCATACAGG	*Eco*RI	56° C

Supplementary Table 6. Primers of the STS markers used in this study.

Marker name	Type	Primer sequence 5'-3' (Forward)	Primer sequence 5'-3' (Reverse)	Annealing temperature
C11837_STS-4	STS	CGTGCCTATTCTGTCTGTACC	CACATACTGACTTTCCTCTCAAA	55° C
C69317_STS	STS	TATGCACAACGGAAGCCTTC	TGCCAATCAATTTCACGAGATCC	55° C

Supplementary Table 7. Primers of the KASP markers used in this study.

Marker name	Type	Primer sequence 5'-3' (Forward)-1	Primer sequence 5'-3' (Forward)-2	Primer sequence 5'-3' (Reverse)
C122784r_KASP	KASP	Gaaggtgaccaagttcatgct GTCAGTTGTCCAAATGCACCA	Gaaggtcggagtcaacggatt GTCAGTTGTCCAAATGCACCt	GGTAACCCCACGAGACGATAA
C67147r_KASP	KASP	Gaaggtgaccaagttcatgct CCGGGACTAATAGGTGTTTTTCTAC	Gaaggtcggagtcaacggatt CCGGGACTAATAGGTGTTTTTCTAT	CATTTGTGGAAGTCGTGGCAA
S747_KASP-7	KASP	Gaaggtgaccaagttcatgct TGCAAATGACTACAAGATGAAGC	Gaaggtcggagtcaacggatt TGCAAATGACTACAAGATGAAGT	TATGTCTGCGCCTACTCACA
S747_KASP-6	KASP	Gaaggtgaccaagttcatgct GAACCAAGAATCCTCACCTCAG	Gaaggtcggagtcaacggatt GAACCAAGAATCCTCACCTCAA	GTTGATAGCGTGGTGTGTCC
C11308_KASP	KASP	Gaaggtgaccaagttcatgct GTCAACGAGATACAAGACGCC	Gaaggtcggagtcaacggatt GTCAACGAGATACAAGACGCG	GGAAGCTCTGAGTAGCATGGT
C137197_KASP	KASP	Gaaggtgaccaagttcatgct CCAAATTAACCAACGCGCCC	Gaaggtcggagtcaacggatt CCAAATTAACCAACGCGCCG	TTGGTTTGGTCTGGTCTGGTT
C2468909_KASP	KASP	Gaaggtgaccaagttcatgct ACTCAAAGGCAATGATCGCG	Gaaggtcggagtcaacggatt CACTCAAAGGCAATGATCGCT	GAAACCCAGAGCGCTTTCATC

Supplementary Table 8. Phenotypes and genotypes of the key recombinants derived from the 803 family segregating for *Sr62*.

Marker	1644	2189	AS_ 8034	AS_ 8043	AS_ 8045	BW_ 22968	BW_ 22981	BW_ 22984	BW_ 23026	BW_ 23033	BW_ 23038	BW_ 23039	BW_ 23042	BW_ 23044
C122784r_KASP	B	A	A	H	B	A	A	B	A	H	H	A	H	H
C11308_KASP	B	A	A	H	B	A	A	B	A	H	H	A	H	H
S741_ KASP-7	B	A	A	H	B	H	A	B	A	H	H	A	H	H
C69317_ STS-1	B	A	H	H	H	H	H	B	A	A	H	H	A	A
Sr62 (phenotype)	R	S	seg	seg	seg	seg	seg	R	S	S	seg	seg	S	S
C03246_CAPS	B	A	H	H	H	H	H	H	A	A	H	H	A	A
S741_KASP-6	B	A	H	H	H	H	H	H	A	A	H	H	A	A
C67147r_ KASP	B	A	H	B	H	H	H	H	H	A	B	H	A	A

R, resistant
S, susceptible
seg, segregating

Supplementary Table 9. Mutations detected in genes in the *Sr62* mapping interval identified by RNA-Seq mapping.

Genotype	Remorin	WAK	WTK	NLR	WTK5	50S-RP	TOE1-B1
12a	Low expression	Low expression			G>A, Asp>Asn		
44d	Low expression	Low expression			G>A, Glu>Lys		
263g	Low expression	Low expression			G>A, Ala>Thr		
353g	Low expression	Low expression			G>A, Gly>Arg		
905c	Low expression	Low expression			C>T, Thr>Met		
734e	Low expression	Low expression			G>A, Asp>Asn; G>A, Gly>Ser		
896d	Low expression	Low expression			G>A, Trp>stop		
119d	Low expression	Low expression	G>A, Gly>Arg				
267d	Low expression	Low expression					
1298e	Low expression	Low expression	C>T, Pro>Leu	C>T, Ala>Ala			
743a	Low expression	Low expression					
190d	Low expression	Low expression					
200h	Low expression	Low expression		C>T, Arg>stop			
1101a	Low expression	Low expression					
Wildtype	Low expression	Low expression					
p-value being candidate	NA	NA	0.664	0.660	8.20E-05	1.000	1.000

Supplementary Table 10. Primers used for making *Sr62* binary construct used in this study.

Marker name	Primer sequence 5'-3' (Forward)	Primer sequence 5'-3' (Reverse)	Annealing temperature
Sr62_P1	TCACTGAgcggccgcaGGTGGT-GATTAGATAACGGTAATGACT	AGTCAGTGgaattcGAGTGA-CTTTAACCTGTGACGGACA	63C (5 cycles)+ 68C (25 cycles)
Sr62_P2	AGTCAGTGgaattcCGTCCATC-CCTCTAACCCCACTT	CTGACTgtttaaacAACACCG-TGACATCTCCATACAGTT	63C (5 cycles)+ 68C (25 cycles)

Supplementary Table 11. Stem rust isolates/races used in the study.

Entry	Isolate	Race	From country
1	04KEN156/04	TTKSK	Kenya
2	13ETH18-1	TKTTF	Ethiopia
3	UK-01	TKTTF	United Kingdom
4	69MN399	QTHJC	USA
5	IS#2079	TKTSC	Israel
6	IS#2127	TTTTF	Israel
7	IS#2135	TTTTC	Israel
8	KE184a/18	TTKTT	Kenya
9	ET11a/18	TKTTF	Ethiopia
10	IT200a/18	TKKTF	Italy
11	IT16a/18	TTRTF	Italy

Supplementary Tables 12. SIFT scores (probabilities) for the predicted Sr62 EMS-induced amino-acid substitutions being tolerated or in-tolerated.

Mutant	Substitution position	Substitution	Probability	Tolerated / In-tolerated
44d	10	E>K	0.07	Tolerant
353g	57	G>R	0.00	In-tolerated
12a	177	D>N	0.00	In-tolerated
263g	222	A>T	0.14	Tolerated
896d	333	W>Stop	NA	NA
734e	405	D>N	0.66	Rolerated
734e	539	G>S	0.00	In-tolerated
905c	617	T>M	0.05	Tolerated

Supplementary Table 13. Percentage nucleotide coding sequence and predicted amino acid identity between *Sr62* (*WTK-A*), *WTK-B* and *Pm24*.

**Coding DNA sequence**				**Amino acid sequence**		
Identity	*WTK5*	*WTK*		Identity	*WTK5*	*WTK*
Pm24	0.624	0.927		*Pm24*	0.527	0.878
WTK5		0.630		*WTK5*		0.536
