# Supplementary material for: Aegilops sharonensis genome-assisted identification of stem rust resistance gene Sr62
Source: Nat Commun. 2022 Mar 25;13:1607. doi: 10.1038/s41467-022-29132-8 (PMC8956640; doi:10.1038/s41467-022-29132-8)
Supplement: Supplementary file 2 — Description of Additional Supplementary Files [file 41467_2022_29132_MOESM2_ESM.pdf]

Description of Additional Supplementary Files

File name: Supplementary Data 1

Description: 5 SNPs and WGS contig source information for Sequenom markers.

File name: Supplementary Data 2

Description: Reads per kilobases (RPK) and transcript per million (TPM) of Sr62 mutants.

File name: Supplementary Data 3

Description: Identification of a conserved amino acid (amino acid 57, in bold) in the binding site of Sr62.

File name: Supplementary Data 4

Description: Identification of a conserved amino acid (amino acid 177, in bold) in the active site of Sr62.

File name: Supplementary Data 5

Description: Presence of Sr62 homologues in plant genomes.
