# Supplementary material for: Aegilops sharonensis genome-assisted identification of stem rust resistance gene Sr62
Source: Nat Commun. 2022 Mar 25;13:1607. doi: 10.1038/s41467-022-29132-8 (PMC8956640; doi:10.1038/s41467-022-29132-8)
Supplement: Supplementary file 8 — Reporting Summary [file 41467_2022_29132_MOESM8_ESM.pdf]

nature research

Corresponding author(s): Brande B. H. Wulff
Brian J. Steffenson

Last updated by author(s): 3 February 2022

Reporting Summary

Nature Research wishes to improve the reproducibility of the work that we publish. This form provides structure for consistency and transparency in reporting. For further information on Nature Research policies, see our Editorial Policies and the Editorial Policy Checklist.

Statistics

For all statistical analyses, confirm that the following items are present in the figure legend, table legend, main text, or Methods section.

n/a	Confirmed	
☐	☒	The exact sample size (*n*) for each experimental group/condition, given as a discrete number and unit of measurement
☒	☐	A statement on whether measurements were taken from distinct samples or whether the same sample was measured repeatedly
☐	☒	The statistical test(s) used AND whether they are one- or two-sided<br>*Only common tests should be described solely by name; describe more complex techniques in the Methods section.*
☒	☐	A description of all covariates tested
☐	☒	A description of any assumptions or corrections, such as tests of normality and adjustment for multiple comparisons
☒	☐	A full description of the statistical parameters including central tendency (e.g. means) or other basic estimates (e.g. regression coefficient) AND variation (e.g. standard deviation) or associated estimates of uncertainty (e.g. confidence intervals)
☐	☒	For null hypothesis testing, the test statistic (e.g. F, t, r) with confidence intervals, effect sizes, degrees of freedom and P value noted<br>*Give P values as exact values whenever suitable.*
☒	☐	For Bayesian analysis, information on the choice of priors and Markov chain Monte Carlo settings
☒	☐	For hierarchical and complex designs, identification of the appropriate level for tests and full reporting of outcomes
☒	☐	Estimates of effect sizes (e.g. Cohen's *d*, Pearson's *r*), indicating how they were calculated

Our web collection on statistics for biologists contains articles on many of the points above.

Software and code

Policy information about availability of computer code

Data collection	No software was used to collect the stem rust phenotype data.
Data analysis	Genome assembly:<br>TRITEX (Monat et al. 2019: Genome Bio. 20, 284)<br>BBMerge (Bushnell et al., 2017: PLOS ONE 12: e0185056)<br>BFC (Li, H. 2015: Bioinformatics 31: 2885-2887)<br>Minia3 (Chikhi & Rizk, 2016: Bioinformatics. 32: i201–i208)<br>SOAPDenovo (Luo et al., 2012: Gigascience 1: 18)<br>GapCloser (Luo et al., 2012: Gigascience 1: 18)<br>BUSCO (v4.06, viridiplantae orthodb10)<br><br>Sequenom marker design:<br>BWA v0.7.17<br>BCFtools v1.2<br>MassARRAY software v3.1<br><br>STS/CAPS/KASP primer design:<br>http://bioinfo.ut.ee/primer3-0.4.0/<br><br>Annotation of genes in mapping interval:<br>CLC Assembly Cell v5.0.0<br>Trimmomatic v0.32

Hisat2 v2.1.0 (default parameters)
SAMtools v.1.8
IGV v2.8.13
Geneious v7.0

GBS plotting:
bwa mem v0.7.12 (default parameters)
SAMtools v.0.1.9
Custom scripts: https://github.com/steuernb/GBS_introgression_line_analysis

Mutant RNA mapping:
Trimmomatic v0.208
Hisat2 v2.1.0 (default parameters)
SAMtools v.1.8
IGV v2.8.13
SIFT (v6.2.1, https://sift.bii.a-star.edu.sg/www/SIFT_seq_submit2.html)

Phylogenetic tree construction:
https://www.ebi.ac.uk/Tools/msa/clustalo/
https://itol.embl.de/
Protein scan: hmmscan v3.1b2

3D modelling:
http://www.sbg.bio.ic.ac.uk/~phyre2/html/page.cgi?id=index
CCP4MG v2.10.11

For manuscripts utilizing custom algorithms or software that are central to the research but not yet described in published literature, software must be made available to editors and reviewers. We strongly encourage code deposition in a community repository (e.g. GitHub). See the Nature Research guidelines for submitting code & software for further information.

Data

Policy information about availability of data

All manuscripts must include a data availability statement. This statement should provide the following information, where applicable:

- Accession codes, unique identifiers, or web links for publicly available datasets
- A list of figures that have associated raw data
- A description of any restrictions on data availability

The raw genomic sequence reads of Ae. tauschii accession 1644 have been deposited in the European Nucleotide Archive (ENA) under project number PRJEB40322.

The genome assembly has been deposited in the ENA under project number PRJEB40049.

The RNA-Seq data for AS_1644, the full-length AS_1644 cDNA library, and Zahir-1644 introgression line wild type and its 14 mutants has been deposited in NCBI under project number PRJEB47173.

The full-length AS_1644 cDNA library sequencing assembly has been deposited in eDAL! under project number https://doi.org/10.5447/ipk/2021/21.

The Zahir-1644 and mutant GBS data have been deposited in ENA under project number PRJEB46949.

The Sr62 sequence has been deposited in GenBank under accession number MZ826707.

The following public databases/datasets were used in the study:

Chinese Spring reference genome (IWGSC, 2018)
Gramene: http://www.gramene.org/#
BLAST https://blast.ncbi.nlm.nih.gov/Blast.cgi?PROGRAM=blastx&PAGE_TYPE=BlastSearch&LINK_LOC=blasthome
(non-redundant protein sequence-nr)
Taxonomy Browser: https://www.ncbi.nlm.nih.gov/Taxonomy/Browser/wwwtax.cgi?id=1437183

Field-specific reporting

Please select the one below that is the best fit for your research. If you are not sure, read the appropriate sections before making your selection.

☒ Life sciences ☐ Behavioural & social sciences ☐ Ecological, evolutionary & environmental sciences

For a reference copy of the document with all sections, see nature.com/documents/nr-reporting-summary-flat.pdf

Life sciences study design

All studies must disclose on these points even when the disclosure is negative.

Sample size	No sample size calculation was chosen. For the initial Sr62 linkage mapping, 49 plus 192 plants from the cross between accession 1644 (resistant) and 2189 (susceptible) were used to map Sr62. Based on a genetic map size of 631 cM (Yu et al. 2017), this is sufficient to map a gene to a defined interval on a chromosome arm. For high resolution mapping, 4,638 plants (9,276 gametes) were used. This is number represents what we could practically handle while striving to reduce the genetic size of the interval as much as possible.
Data exclusions	We only used plants to map Sr62 for which we obtained good quality DNA. Samples with one or both flanking markers missing were not included in the screen for recombinants.
Replication	Since Sr62 was mapped in a segregating population, we could not replicate these phenotypes.
Randomization	The stem rust tests were not randomized as the infection type were taken as a qualitative trait (either homozygous susceptible, homozygous resistant, or segregating) for linkage mapping. No quantitative analysis of the phenotype was conducted.
Blinding	In essence, you could argue that blinding was applied for the initial mapping as the phenotypes of the recombinants used to map Sr62 were obtained after the genotypes. For the transgenics, and other segregating populations used in this study, the genotypes were obtained after the phenotypes.

Reporting for specific materials, systems and methods

We require information from authors about some types of materials, experimental systems and methods used in many studies. Here, indicate whether each material, system or method listed is relevant to your study. If you are not sure if a list item applies to your research, read the appropriate section before selecting a response.

Materials & experimental systems

n/a	Involved in the study
☒	☐ Antibodies
☒	☐ Eukaryotic cell lines
☒	☐ Palaeontology and archaeology
☒	☐ Animals and other organisms
☒	☐ Human research participants
☒	☐ Clinical data
☒	☐ Dual use research of concern

Methods

n/a	Involved in the study
☒	☐ ChIP-seq
☒	☐ Flow cytometry
☒	☐ MRI-based neuroimaging
